# An integrated genetic, genomic and systems approach defines gene networks regulated by the interaction of light and carbon signaling pathways in Arabidopsis

**DOI:** 10.1186/1752-0509-2-31

**Published:** 2008-04-04

**Authors:** Karen E Thum, Michael J Shin, Rodrigo A Gutiérrez, Indrani Mukherjee, Manpreet S Katari, Damion Nero, Dennis Shasha, Gloria M Coruzzi

**Affiliations:** 1 Department of Biology, New York University, New York, NY, 10003, USA; 2 Department of Biology, Messiah College, Grantham, PA, 17027, USA; 3 Departamento de Genética Molecular y Microbiología, Pontificia Universidad Católica de Chile. Alameda 340. 8331010. Santiago, Chile; 4 Courant Institute of Mathematical Sciences, New York University, New York, NY, 10003, USA

## Abstract

**Background:**

Light and carbon are two important interacting signals affecting plant growth and development. The mechanism(s) and/or genes involved in sensing and/or mediating the signaling pathways involving these interactions are unknown. This study integrates genetic, genomic and systems approaches to identify a genetically perturbed gene network that is regulated by the interaction of carbon and light signaling in Arabidopsis.

**Results:**

Carbon and light insensitive (*cli*) mutants were isolated. Microarray data from *cli186 *is analyzed to identify the genes, biological processes and gene networks affected by the integration of light and carbon pathways. Analysis of this data reveals 966 genes regulated by light and/or carbon signaling in wild-type. In *cli186*, 216 of these light/carbon regulated genes are misregulated in response to light and/or carbon treatments where 78% are misregulated in response to light and carbon interactions. Analysis of the gene lists show that genes in the biological processes "energy" and "metabolism" are over-represented among the 966 genes regulated by carbon and/or light in wild-type, and the 216 misregulated genes in *cli186*. To understand connections among carbon and/or light regulated genes in wild-type and the misregulated genes in *cli186*, the microarray data is interpreted in the context of metabolic and regulatory networks. The network created from the 966 light/carbon regulated genes in wild-type, reveals that *cli186 *is affected in the light and/or carbon regulation of a network of 60 connected genes, including six transcription factors. One transcription factor, HAT22 appears to be a regulatory "hub" in the *cli186 *network as it shows regulatory connections linking a metabolic network of genes involved in "amino acid metabolism", "C-compound/carbohydrate metabolism" and "glycolysis/gluconeogenesis".

**Conclusion:**

The global misregulation of gene networks controlled by light and carbon signaling in *cli186 *indicates that it represents one of the first Arabidopsis mutants isolated that is specifically disrupted in the integration of both carbon and light signals to control the regulation of metabolic, developmental and regulatory genes. The network analysis of misregulated genes suggests that *CLI186 *acts to integrate light and carbon signaling interactions and is a master regulator connecting the regulation of a host of downstream metabolic and regulatory processes.

## Background

Carbon and light are two important and interdependent signals that regulate plant growth and development. One mechanism by which these signals exert their effects on plants is through their ability to affect the expression of a large number of genes through signal transduction cascades. While much is known about how plants respond to and transduce light signals [1-6], less is known about the perception and transduction of carbon signals [7-13]. Moreover, while carbon and light signaling pathways influence one another via "crosstalk" [13-18], nothing is yet known about the molecular components that might link these two signaling pathways.

Microarray studies have been used to investigate the integration of multiple inputs such as carbon and nitrogen [19-21], carbon and hormones (i.e. abscisic acid) [22] as well as carbon and circadian rhythms [23]. These studies demonstrate that the carbon-regulated genes are representative of a diverse range of biological processes, such as metabolism (carbohydrate, amino acid and fatty acid and lipid), energy, protein synthesis and stress (heat-shock proteins), among others. Microarray studies have also been used to investigate the genes and encoded biological processes that are subject to a significant degree of regulation by light and carbon interactions in light-grown Arabidopsis seedlings [18]. Results from our previous study revealed that the majority of genes analyzed (63%) showed regulation by light and carbon interactions. Furthermore, functional category analysis revealed that genes in the biological process "metabolism", were significantly controlled by the interaction of carbon and light in light-grown plants [18]. Other studies of carbon and light interactions have shown synergistic or antagonistic relationships between light and carbon signaling on a gene-by-gene basis [17]. For example, genes related to photosynthesis are strongly induced by light, yet repressed by carbon treatment (*e.g*. chlorophyll a/b binding protein, plastocyanin, small subunit of rubisco) [7]. For other genes, the effects of carbon are distinct in the presence or absence of light. For example, a number of genes involved in N-assimilation (*e.g*. glutamine synthetase 2) are induced by carbon in dark-adapted plants [7,17,24,25], but are repressed by carbon in light-treated plants [17]. More specific interactions between carbon and light signaling have been observed by the ability of carbon to suppress a far-red/phytochrome A-specific, light-induced block of greening [14]. Here, carbon may antagonize or suppress a phytochrome A signaling pathway(s).

A number of studies have used genetic approaches to identify genes involved in light or carbon signaling. Some genetic screens have focused on the isolation of Arabidopsis mutants involved in carbon signaling [7,26-28] or in light sensing and signaling [2,3,6]. Several of these genetic studies have used light signaling mutants to test the influence of carbon treatments on phytochrome signal transduction pathways [14-16]. Thus far, there have been no reports of the isolation of mutants identifying components that mediate or mechanisms involved in the signaling interactions between carbon and light signaling.

In this study, a carbon and light insensitive (*cli186*) mutant is identified and its molecular defects characterized on a genome-wide scale, using a multinetwork approach to identify the genes, biological processes and regulatory/metabolic networks affected in the *cli186 *mutant. The multinetwork analysis of microarray data reveals connections between metabolic and regulatory networks that are perturbed in the *cli186 *mutant that could only be discovered via this integrated network analysis. This combined genetic, genomic and network analyses of a carbon and light insensitive (*cli186*) mutant described herein, identifies *CLI186 *as a putative major regulatory gene that integrates carbon and light signaling to control a downstream network of metabolic and regulatory genes in Arabidopsis.

## Results

### A positive genetic selection for carbon and light insensitive mutants (*cli*)

**Figure 1:**
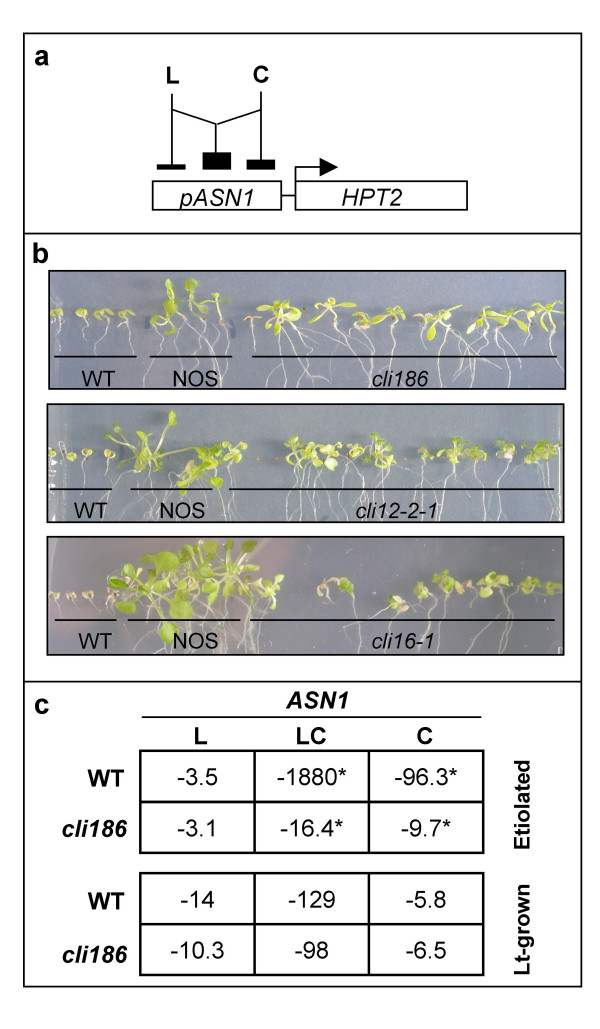
Reporter construct used for mutant screen and verification of *ASN1 *gene expression in *cli186*. (a) Schematic representation showing regulation of the *ASN1-HPT2 *reporter construct used to select for carbon and light insensitive (*cli*) plants. A 148-bp region of the *ASN1 *promoter from pea was placed upstream of the hygromycin phosphotransferase gene, *HPT2*. *ASN1 *is transcriptionally repressed by sucrose and by light independently, where sucrose and light together have a synergistic repressive effect. (b) Three mutagenized lines, *cli186*, *cli12-2-1 *and *cli16-1 *that exhibit hygromycin-resistance when screened on 0.5% sucrose in L/D cycling conditions. Controls consist of a 'wild-type' (WT) unmutagenized line containing the *ASN1-HPT2 *transgene and a transgenic line (NOS) containing the *HPT2 *transgene driven by a NOS promoter, allowing for constitutive expression of the *HPT2 *gene. (c) Fold-repression as determined via Q-PCR of *ASN1 *in WT and *cli186 *plants. Seven day old etiolated seedlings were subject to four treatments: -C-L, +C-L, -C+L and +C+L. Fold-repression of *ASN1 *was determined by comparing all treatments against their respective backgrounds of -C-L. Asterisks indicate a significant difference between WT and *cli186 *in expression based on a t-test, p > 0.05.

In previous studies, it has been shown that the asparagine synthetase (*ASN1*) gene in plants is transcriptionally repressed by transient treatments with sucrose and/or light, where both light and sucrose together have a synergistic repressive effect [24,29,30]. To identify genetic components involved in the integration of light and carbon signaling, the *ASN1 *promoter was used in a positive genetic selection, designed to identify mutants defective in the transcriptional repression of the *ASN1 *gene by both light and carbon. An Arabidopsis line was created that contained a transgene in which a 148-bp region of the *ASN1 *promoter from pea was placed upstream of the hygromycin phosphotransferase gene (*HPT2*) [30,31] (Figure 1a). As light and carbon treatments repress expression of ASN1 in the light on carbon-containing media, the *ASN1*::*HPT2 *lines whose growth was suppressed under these conditions were selected for mutagenesis. A similar positive genetic selection scheme using the reporter gene construct, *CAB3*::*HPT2 *was used in the isolation of the dark overexpression of cab mutants (*doc*), which identified genes controlling expression of the *CAB3 *gene [32].

Seeds from one transgenic *ASN1*::*HPT2 *line [31] were mutagenized using either fast neutron irradiation or EMS (see Methods). M2 mutagenized seeds were germinated and grown in the light on media containing 1% sucrose and 15 μg/ml hygromycin. Putative carbon and light insensitive (*cli*) mutants showing a loss of *ASN1*::*HPT2 *transgene repression by light and carbon treatments were selected, based on their degree of resistance to hygromycin (Figure 1b). Hygromycin-resistance was determined by increased root length, greening of primary and secondary leaves and overall enhanced growth over a period of three weeks, when compared to the un-mutagenized wild-type transgenic *ASN1*::*HPT2 *parental line (Figure 1b).

From an initial screen of approximately 20,000 M2 fast-neutron irradiated *ASN1*::*HPT2 *seedlings (isolated from 763 individual M1 lines), one line, *cli186*, retained a consistent hygromycin-resistant phenotype past the M3 generation (Figure 1b). A screen of approximately 23,000 additional M2 seeds from 579 M1 EMS mutagenized *ASN1*::*HPT2 *lines identified 12 additional putative *cli *mutants [33] that retained a consistent hygromycin phenotype beyond the M3 generation, two of which are shown in Figure 1b, *cli12-2-1 *and *cli16-1*.

### *ASN1 *egulation by carbon and light is disrupted in *cli186*

As the *cli *mutant selection was initially based on misregulation of a pea *ASN1*::*HPT2 *transgene, in a secondary screen, it was determined whether the regulation of the endogenous Arabidopsis *ASN1 *gene was also aberrant in response to light and carbon treatments. Plants at two different stages of development were analyzed: etiolated and light-grown. For etiolated studies, plants were grown in the presence or absence of 1% sucrose for seven days in the dark, after which half were maintained in the dark and the other half were illuminated with white light (70 μEin m^-2^s^-1^) for an additional three hours. For light-grown studies, plants were grown in 16-h light/8-h dark cycling conditions for 14 days on sucrose-containing (1%) media and were thereafter transferred to fresh media containing either no sucrose or 1% sucrose and placed in the dark or white light (70 μEin m^-2^s^-1^) for an additional three hours. The change of endogenous *ASN1 *transcript levels in response to various transient treatments of light and carbon (+L-C; +L+C; -L+C; -L-C) were compared in wild-type and *cli *mutants to confirm light and/or carbon misregulation of the endogenous *ASN1 *gene. Quantification of endogenous levels of *ASN1 *mRNA (see section below) in the 13 *cli *mutants revealed misregulation of *ASN1 *mRNA with regard to light and carbon repression, when compared to wild-type (Figure 1c, data not shown for additional *cli *mutants). The *cli*186 mutant exhibited the most dramatic misregulation of *ASN1 *regulation by light and carbon interactions and was thus selected as the major focus of this genomic/network study.

Fold-repression of *ASN1 *mRNA levels by light/carbon treatments is shown for *cli186 *compared to wild-type for light grown versus etiolated seedlings (Figure 1c). When grown in the dark in the presence of 1% sucrose, *ASN1 *levels were repressed 96.3-fold in wild-type. This carbon repression of *ASN1 *was reduced to 9.7-fold in *cli186 *(Figure 1c; C). Therefore, carbon regulation of *ASN1 *in etiolated seedlings is impaired approximately 10-fold in *cli186 *mutants as compared to wild-type plants. Repression of *ASN1 *mRNA levels in response to light only treatments was similar in wild-type and *cli186 *(~3-fold), suggesting that light regulation of *ASN1 *is not impaired in *cli186 *(Figure 1c; L). Intriguingly, the interaction of light and carbon repression of *ASN1 *is dramatically perturbed in *cli186*, as levels of *ASN1 *mRNA are repressed 1,880-fold in wild-type, compared to 16.4-fold in *cli186 *(Figure 1c, LC). Thus, the regulation of *ASN1 *by the interaction of light-and-carbon was significantly disrupted (114.5-fold) in *cli186*, compared to wild-type when assayed in etiolated plants.

Fold repression of *ASN1 *mRNA levels by light and carbon treatments was also determined in 14-day old light-grown plants (Figure 1c). When light-grown plants were transiently treated with 1% sucrose in the dark, *ASN1 *mRNA levels were similarly repressed in wild-type (5.8-fold) and in *cli186 *(6.5-fold) (Figure 1c; C). In light only treatments, the fold repression of *ASN1 *mRNA was also similar between wild-type (14-fold) and *cli186 *(10.3-fold). Again there were differences observed in *ASN1 *mRNA levels in plants treated with the combination of light plus carbon in wild-type (129-fold) versus *cli186 *(98-fold). Thus, although not as dramatic as that observed for etiolated seedlings, the regulation of *ASN1 *by the interaction of light-and-carbon remained disrupted in *cli186 *in light-grown seedlings when compared to wild-type.

These results confirm that in wild-type seedlings, the combination of light and carbon together repress *ASN1 *mRNA levels 1,880-fold in etiolated seedlings and 129-fold in light-grown plants, which being more than the sum of the two factors individually in either developmental state is indicative of a synergistic light/carbon interaction. Interestingly, this synergistic interaction of light and carbon in the regulation of *ASN1 *was dramatically lost in the *cli186 *mutant, specifically when monitored in etiolated seedlings (Figure 1c, LC). Thus, investigation of the *cli186 *mutant should aid in elucidating the mechanism(s) underlying the interaction between light and carbon signaling, and how this intersects with loss of carbon only signaling in etiolated plants (Figure 1c, C).

### Genomic microarray analysis of wild-type and *cli186 *seedlings

Microarray analysis combined with systems biology tools was used to determine which genes, biological processes and gene networks are targets of light/carbon signaling interactions in wild-type and which of these networks are disrupted in the *cli*186 mutant. Affymetrix whole genome ATH1 chips were hybridized with cRNA made from RNA isolated from wild-type and *cli*186 etiolated seedlings. Plants were grown for seven days in the dark (etiolated) in the presence or absence of 1% sucrose and subjected to transient light treatments (e.g. three hours of white light versus three hours of additional darkness). Using this general approach, wild-type and *cli*186 plants were subject to four different light and/or carbon treatments (-L+C; +L-C; +L+C; -L-C). Treatment with no light and no carbon (-L-C) acted as a control or baseline treatment, to which all other treatments were compared. Each of two background treatments (i.e. -C-L) were compared with both replicates for each treatment (e.g. +L-C) which provided a four-way comparison for each of the three treatments. The treatments compared to the background will be referenced in the following manner: C = carbon-only (-L+C/-L-C), L = light-only (+L-C/-L-C), LC = light-plus-carbon, (+L+C/-L-C). Genes that responded to the various light and/or carbon treatments (C, LC, or L) were further identified and classified based on parameters assigned to each gene by Microarray Suite 5.0 and using a 'metric' classification system as described in Methods.

Data analysis from gene chips performed on wild-type and *cli186 *mRNA was carried out simultaneously to classify genes based on their regulation by light and/or carbon across all 24 comparisons (4 comparisons/treatment × 3 treatments × 2 genotypes: wild-type and *cli186*). Three filters were applied to the initial dataset to identify the subset of genes for further analysis. To eliminate probes for genes that could not be reliably compared between treatment and baseline due to poor detection from either low expression or aberrant hybridization, Filter 1 was applied to eliminate probes with "absent" detection calls in both treatment and baseline hybridizations. To include probes representing genes that are expressed at a low level, probes whose expression was low but detectable (signal value of 50 or greater) was compared with those probes that were 'absent' in the treatment or the baseline hybridization. Thus, Filter 2 included probes with an "absent" call in treatment or baseline if the "present' probe in the other hybridization had a signal value of 50 or greater. To ensure high reproducibility of genes within our dataset, Filter 3 was used to include genes in which the difference calls in 3 out of 4 comparisons between treatment and baseline in wild-type and mutant were consistent (i.e. "induced", "repressed", "not change") as provided by the Affymetrix Microarray Suite 5.0 software. Thus, genes that performed reproducibly 75% of the time were included in the dataset. When applied to the wild-type and *cli186 *dataset over all 24 comparisons (3 treatments × 2 replicates × 2 controls = 12 treatments for each genotype × 2 genotypes (wt and mutant) = 24 comparisons), these rigorous filtering steps removed probes corresponding to 21,186 genes, leaving 1,624 genes that showed consistent reliability for further analysis. This final filtered dataset of 1,624 genes is thus stringent and reliably reproducible across all the comparisons examined. These 1,624 genes were further analyzed below to determine what patterns of regulation by light and/or carbon were found in wild-type and *cli186 *seedlings.

### Identification of 966 genes regulated by light and/or carbon in wild-type and 216 genes misregulated by light and/or carbon in *cli186*

In order to identify misregulated genes in the *cli186 *mutant, a 'metric' classification system (see Methods) was designed to organize the filtered genes into classes based on their relative expression profiles across all three types of treatments: L, C, LC. The classification system uses a statistical approach to determine those genes that are regulated by light and/or carbon in wild-type, and that show different responses (misregulated) in the corresponding experiments in the *cli186 *mutant. ANOVA analysis was used to compare gene regulation across all three treatments (L-only, LC, C-only), for both wild-type and *cli186*. Multiple testing was addressed by controlling the false discovery rate at 5% as previously described [34]. 658 of the 1,624 "reliable" genes did not show significant differences across any of the three treatments (L, LC or C) when comparing wild-type or *cli186 *responses. In contrast, 966 of the 1,624 genes changed significantly in at least one treatment (L, LC or C) in wild-type or *cli186*, suggesting interactions of light and carbon or an altered response to the light and/or carbon treatments in the *cli186 *mutant. A list of genes and their classes that are light/carbon regulated in wild-type and *cli186 *is included [see Additional file 1]. A list of the 966 light/carbon regulated genes in wild-type and the 216 misregulated genes in *cli186 *is included [see Additional file 2]. Closer inspection of the resulting patterns of regulation revealed 216 genes that were misregulated in *cli186 *in light and/or carbon treatments when compared to wild-type. A total of 900 genes (or 93% of 966 genes) are regulated by all three treatments (L, C and/or LC) in wild-type and exhibit either normal regulation (same as wild-type) OR misregulation by light and/or carbon in *cli186 *(Table 1a, 1c). Of the remaining 66 genes, 22 genes (2.3%) are regulated by LC only, and 21 genes (2.2%) are regulated by L-only and C-only in wild-type (Table 1a). Moreover, 14 genes (1.3%) that are not regulated by L/C in wild-type, are regulated in any one or combination of L and/or C treatments in *cli186 *(Table 1c). In total, of these 966 light/carbon regulated genes in wild-type, 216 are misregulated in *cli186 *in any one or combination of light and/or carbon treatments (Table 1b). Patterns of misregulation observed among the 216 misregulated genes in *cli186 *is included [see Additional file 3].

**Table 1:** Number and percent of (a) L/C regulated genes in WT or (b) L/C misregulated genes in *cli186 *and (c) the comparison of genes misregulated in *cli186 *with their regulation in WT in any one or combination of treatments.

a	Treatment			WT L/C Regulation		b	Treatment			*cli186 *L/C Misregulation	
	L	LC	C	#	%		L	LC	C	#	%
1)	L	-	-	2	0.2	**1)**	**L**	**-**	**-**	**31**	**14.0**
2)	-	-	C	1	0.1	**2)**	**-**	**-**	**C**	**36**	**17.0**
3)	-	LC	-	22	2.3	**3)**	**-**	**LC**	**-**	**91**	**42.0**
4)	L	-	C	21	2.2	**4)**	**L**	**-**	**C**	**3**	**1.4**
5)	L	LC	-	4	0.4	5)	L	LC	-	41	19.0
6)	-	LC	C	2	0.2	6)	-	LC	C	7	3.2
7)	L	LC	C	900	93.0	7)	L	LC	C	7	3.2
8)	-	-	-	14	1.3	8)	-	-	-	0	0

	Total			966	99.9		Total			216	100

		Misregulation of genes observed in *cli186 *in:									
				
c	Regulation of genes observed in WT in:	L (31)	C (36)	LC (91)	L_C (3)	L_LC (41)	LC_C (7)	L_LC_C (7)	WT Regulation (14)		

(2)	L	-	-	-	-	-	-	-	2		
(1)	C	-	1	-	-	-	-	-	-		
(22)	LC	1	-	8	-	-	1	-	12		
(21)	L_C	-	-	10	1	-	-	-	10		
(4)	L_LC	-	2	-	-	-	-	-	2		
(2)	LC_C	-	1	-	-	-	-	-	1		
(900)	L_LC_C	30	28	66	1	41	6	5	723		
(14)	Not regulated	-	4	7	1	-	-	2	-		

The L/C patterns of misregulation in *cli186 *that are perturbed in a L and C interaction are in bold.

Although *cli186 *shows misregulation of genes in a number of light and/or carbon treatments and combinations thereof, the most common pattern of misregulation corresponded to genes specifically misregulated in LC treatments only (91/216 genes or 42% of all misregulated genes, Table 1b). This suggests that *cli186 *may be a mutant defective in a component that integrates light and carbon sensing and/or signaling. Additionally, *cli186 *contains 41/216 genes (19%) that are misregulated in L and LC treatments, 7/216 genes (3.2%) that are misregulated in C and LC treatments, 31/216 genes (14%) that are misregulated in the L-only treatment, and 36/216 genes (17%) that are misregulated in the C-only treatment (Table 1b). Genes that exhibit misregulation in L-only but not in LC treatments, indicate that the carbon signaling pathway antagonizes the light pathway, thus an interaction between light and carbon pathways is present. A similar rationale is applied to those genes that are misregulated in the C-only treatment, but not in LC treatments. Thus, of the 216 misregulated genes in the *cli186 *mutant, 161 genes or 75% (31 genes in L only; 36 genes in C only; 91 genes in LC; 3 genes in both L only and C only) exhibit misregulation specifically in response to an interaction between light and carbon signaling, further supporting the hypothesis that *cli186 *is defective in a component that integrates light and carbon sensing and/or signaling.

To complement and further validate the 'metric' classification system analysis, an ANOVA analysis was used determine the genes regulated by either C and/or L in wild-type and the *cli186 *mutant (see Methods). This 3-way ANOVA was carried out with three dichotomous categorical variables (Carbon, Light and Genotype). The p-values were then corrected for multiple testing using a FDR correction at 5%. Of the 1,625 genes comprising the filtered dataset, we find 1,263 regulated genes and of those, 308 are misregulated in the *cli186 *mutant [see Additional file 4]. There is a significant overlap of genes among the two analysis methods employed. For example, 924 regulated genes and 156 misregulated genes are shared among the two datasets [see Additional file 4]. As this 3-way ANOVA analysis provided similar results as the 'metric' classification system, we carried out all further analyses in this study using the 966 regulated genes and 216 *cli186 *misregulated genes obtained from the 'metric' classification analysis, a method previously used for the classification of microarray datasets [18,19].

### Genes in the biological processes energy and metabolism are regulated by light and/or carbon in wild-type but misregulated in *cli186*

The 966 L/C regulated genes in wild-type and the 216 L/C misregulated genes in *cli186 *were categorized using functional classification terms to determine which biological processes are significantly over-represented in this group of genes. Functional assignments of genes were based on annotations provided by the Munich Information Center for Protein Sequences (MIPS) *Arabidopsis thaliana *database [35,36]. The over-representation of MIPS terms in the gene lists, "L/C Regulated (966)", "L/C Misregulated (216)" were compared to the entire Arabidopsis genome using the BioMaps tool for visualization and statistical analysis, using a p value ≤ 0.05 [37,38].

Table 2a shows the MIPS functional categories over-represented in the 966 L/C regulated genes in wild-type and those over-represented in the list of 216 L/C misregulated genes from *cli186*. Of the 29 primary level MIPS functional categories, "Metabolism" and "Energy" are two significantly over-represented terms among the 966 L/C regulated genes in wild-type (Table 2a). In secondary and tertiary functional categories under "Metabolism" and "Energy", the processes that are significantly over-represented among the 966 L/C regulated genes in wild-type include, "C-compound and carbohydrate metabolism", "glycolysis and gluconeogenesis" and "photosynthesis" (Table 2a). Interestingly, the functional categories "Energy", "glycolysis and gluconeogenesis" and "photosynthesis" are over-represented among both the 966 L/C regulated genes in wild-type and the 216 L/C misregulated genes in *cli186 *(Table 2a). The categories that are over-represented only among the 216 L/C misregulated genes in *cli186 *include, "amino acid metabolism", "nitrogen and sulfur metabolism", "respiration", "aerobic respiration", "plastid" and "chloroplast" (Table 2a). These findings indicate that the biological processes mentioned above that are misregulated in *cli186 *are normally regulated by L, LC and C in wild-type seedlings. This validates that the positive genetic selection for mutants impaired in light/carbon repression of *ASN1::HPT2 *enabled us to isolate an Arabidopsis mutant that affects the light/carbon regulation of genes involved in metabolism and other genes in related energy pathways.

**Table 2:** Biological processes over-represented among (a) all the 966 L/C regulated genes in WT and 216 L/C misregulated genes in *cli186*, (b) the 542 L/C regulated genes in WT and the 92 L/C misregulated genes in *cli186 *that are connected in a network and (c) the 424 L/C regulated genes in WT and the 124 L/C misregulated genes in *cli186 *that are not connected in a network.

		a ALL compared to genome		b IN Network compared to multinetwork		c NOT in network compared to genome	
		
		WT	*cli186*	WT	*cli186*	WT	*cli186*
		
Numerical Category	MIPS Funcational Category	L/C regulated (966)	L/C misregulated (216)	L/C regulated (542)	L/C misregulated (92)	L/C regulated (424)	L/C misregulated (124)
01.	METABOLISM	0.00163	-	-	-	-	-
01.01	amino acid metabolism	-	0.0315	-	-	-	-
01.02	nitrogen and sulfur metabolism	-	0.01365	-	-	-	-
01.02.01	nitrogen and sulfur utilization	-	-	-	-	-	-
01.05	C-compound/carbohydrate metabolism	0.04471	-	-	-	-	-
02.	ENERGY	**1.02E-07**	**2.01E-07**	-	-	-	0.00042
02.01	glycolysis/gluconeogenesis	**0.00175**	**0.00151**	-	-	-	-
02.13	respiration	-	0.00119	-	-	-	4.71E-05
02.13.03	aerobic respiration	-	0.02291	-	-	**0.00034**	**0.00264**
02.30	photosynthesis	**8.16E-05**	**2.53E-08**	-	7.00E-03	**0.00076**	**0.00316**
10.	CELLULAR COMMUNICATION/SIGNAL TRANSDUCTION	-	-	-	-	-	-
10.05	transmembrane signal transduction	-	-	-	-	-	-
10.05.02	non-enzymatic receptor mediated signaling	0.02932	-	-	-	-	-
10.05.02.30	ion channel mediated signaling pathway	0.02932	-	-	-	-	-
30.	CONTROL OF CELLULAR ORGANIZATION	-	-	-	-	-	-
30.26	plastid	-	8.63E-05	-	-	-	1.35E-05
30.26.03	chloroplast	-	8.63E-05	-	-	-	1.35E-05

This table shows the functional categories (funcats) annotated by MIPS [35] that respond to light and/or carbon among the 966 L/C regulated genes in wild-type (WT) and 216 L/C misregulated genes in *cli186*. The funcats are arranged according to their hierarchy as indicated by their numerical category. P-values to determine the significance of the funcats among the subsets of genes are shown in the columns labeled, 'L/C Regulated' and 'L/C Misregulated' in panels (a), (b) and (c). The p-values for the funcats that are significantly over-represented among both the L/C regulated genes in wild-type and the L/C misregulated genes in *cli186 *are in bold in panels (a), (b) and (c). The p-values for the funcats that are in normal text indicate those funcats that show over-representation among the 'L/C Regulated' or the 'L/C Misregulated' groups of genes in all panels, (a), (b) and (c). Dashes indicate no significant representation of the funcat among the groups of genes. Over-representation of MIPS terms in the gene lists were compared to the entire Arabidopsis genome (a), (c) or to the Arabidopsis multinetwork (b) using the BioMaps tool for visualization and statistical analysis, using a p value ≤ 0.05 [37, 38].

### L/C regulated genes in wild-type form a highly connected and complex metabolic and regulatory network that is disrupted in the *cli186 *mutant

We next analyzed the microarray data in a multinetwork context to accomplish the following three goals: 1) To understand how the L/C regulated genes (966 genes) in wild-type are connected to each other from a network perspective, 2) to help understand/identify hubs that may control such a large number of genes in concert, and 3) to determine how the *cli186 *mutation perturbs this network. Integrated metabolic and regulatory networks were created using an Arabidopsis multinetwork tool developed by Gutierrez et al. (2007) [[21]; Methods]. This multinetwork allowed us to use a systems biology approach to identify the metabolic and regulatory interactions affected by the different treatments used and to see how these interactions are changed or perturbed in the *cli186 *mutant. All 966 L/C regulated genes in wild-type were used to query this Arabidopsis multinetwork. Of the 966 L/C regulated genes in wild-type, 542 genes were present in the highly connected multinetwork [see Additional file 5]. The remaining 424 genes are not yet represented by connections in the multinetwork database. To determine if the presence and connectivity of the 542 genes in this L/C network are significant, 966 genes were selected at random from the Arabidopsis genome 10,000 times, and asked how many times a network of 542 connected genes or more could be recapitulated (see Methods). In no cases out of 10,000 randomizations, was a connected network of 542 genes or more recapitulated when randomly selecting 966 genes from the Arabidopsis genome (p < 0.0001). Thus, a subset of 542 L/C regulated genes from wild-type form a significantly connected component of the L/C interaction network.

With the L/C network of 542 genes in place for wild-type Arabidopsis, the genes in this network that are misregulated in *cli186 *were identified, so as to identify the sub-networks and biological processes disrupted by the *cli186 *mutation [see Additional file 5]. Of the 216 L/C misregulated genes in *cli186*, 92 genes are present in the large connected wild-type L/C multinetwork of 542 genes. Again, the significance of the degree of presence (i.e. the overlap of 92 genes) and connectivity of the L/C misregulated genes in the large wild-type multinetwork was determined (see Methods). Both the presence of and connectivity among the 92 L/C misregulated genes in *cli186 *within the wild-type multinetwork is significant (presence: p < 10^-4^, connectivity: p = 0.0418).

To gain an overview of the biological processes that are represented among the 542 connected genes (out of 966) in the L/C gene network in wild-type and the subset of 92 (out of 216) L/C misregulated genes also present in this network, a funcat (functional category) analysis using the MIPS annotations was carried out on these lists of genes, "L/C Regulated (542)" and "L/C Misregulated (92)" (Table 2b). Over-representation of MIPS terms in the gene lists, "L/C Regulated (542)" and "Mis-regulated (92)" were compared to the 6,179 gene nodes represented in the Arabidopsis multinetwork [21] using the BioMaps tool for visualization and statistical analysis, using a p value ≤ 0.05 [37,38]. This analysis revealed that the sub-funcat, "photosynthesis" is the only over-represented term among the L/C misregulated genes in *cli186 *(Table 2b). The over-representation of only one sub-funcat is found because the 6,179 gene nodes from the Arabidopsis multinetwork [21] was used as the background to determine over-represented funcats within the gene lists, "L/C Regulated (542)" and "L/C Misregulated (92)". As the Arabidopsis multinetwork is not complete, there may be a bias of biological processes represented within the multinetwork. Thus, using the Arabidopsis multinetwork [21] instead of the entire Arabidopsis genome as a background for comparison of over-represented terms among the lists of genes, "L/C Regulated (542)" and "Misregulated (92)" accounts for this possible bias.

Although the current version of the Arabidopsis multinetwork is extensive, containing 6,176 gene nodes, 1,459 metabolite nodes and 230,900 interactions, it is not complete. Thus, many genes and their interacting partners are not represented in the multinetwork. To determine which biological processes are represented among those genes that are not represented in the Arabidopsis multinetwork, a MIPS functional category analysis was carried out (Table 2c). This analysis revealed that genes in the biological processes, "respiration" and "aerobic respiration" are over-represented in the genes not present in the multinetwork, which include the 424 L/C regulated genes in wild-type and the 124 L/C misregulated genes in *cli186*. Interestingly, the biological processes, "Energy", "photosynthesis", "plastid" and "chloroplast" are over-represented among the 124 L/C misregulated genes in *cli186*. Thus, the biological processes, "respiration" and "aerobic respiration" are additional processes that are also regulated by L/C in wild-type and misregulated by L/C in *cli186*, but not yet defined by the network analysis. Furthermore, the funcats "Energy", "photosynthesis", "plastid" and "chloroplast" are processes that are L/C misregulated in *cli186*, but not yet defined by the network analysis.

### Supernode network summarizes multinetwork of L/C regulated processes in wild-type and L/C misregulated processes in *cli186*

**Figure 2:**
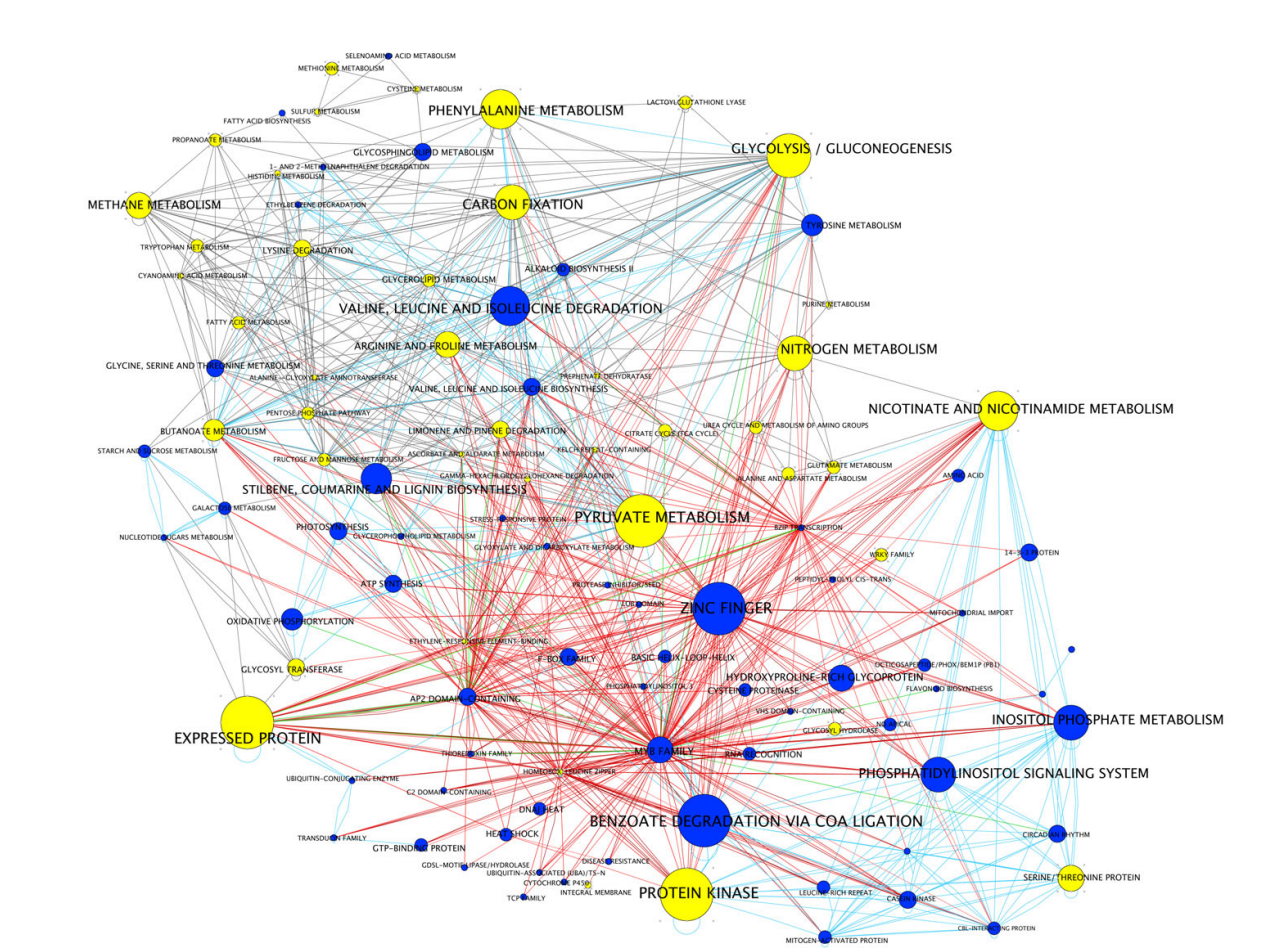
A supernode network summarizes the biological processes regulated in wild-type and misregulated in *cli186 *by L/C treatments. Supernodes are created by collapsing genes into a category according to both their metabolic pathways and the first two words of their gene annotation. This supernode network was created from the large multinetwork analysis that contains the 966 L/C regulated genes in wild-type [see Additional file 6]. Blue nodes represent biological processes that contain genes that are under wild-type L/C regulation in wild-type and *cli186*. Yellow nodes represent biological processes in which at least one gene shows L/C misregulation in *cli186*. The size of the node indicates the number of genes within that particular biological process. Nodes are connected by either metabolic (grey lines), DNA:protein (red lines = positive correlation; green lines = negative correlation) or protein:protein (blue lines) interactions.

To gain an understanding of how the various processes regulated by L/C in wild-type seedlings are connected to each other, and to determine which of these processes are perturbed by the *cli186 *mutation, a supernode network was created (Figure 2). Supernodes are created by collapsing multiple genes from a multinetwork into a single node, according to their annotation. For example, all genes annotated to the glycolysis/gluconeogenesis pathways are summarized in the network with a single node labeled "glycolysis/gluconeogenesis". The size of the node is proportional to the number of genes annotated to the corresponding node label. Gene edges from the large multinetwork are transferred to the supernode, and are summarized in the final supernode network by a single edge type between supernodes. The connectivity of the nodes are supported by edges that may be metabolic (grey line), protein-DNA (red line = positive correlation; green line = negative correlation) or protein-protein (blue dashed line) interactions or combinations thereof. Hence, this supernode analysis provides a summary of the biological processes that are L/C regulated in wild-type and those that are perturbed by the *cli186 *mutation and the connections between these processes.

The supernode network (Figure 2, blue nodes), shows that the supernodes containing the largest number of genes that are under regulation by L/C interactions in both wild type and *cli186 *include: "zinc finger", "benzoate degradation via CoA ligation", "valine, leucine, isoleucine degradation", "stilbene, coumarine and lignin biosynthesis", "phosphatidylinositol signaling system" and "inositol phosphate metabolism". Those processes that contain the largest number of genes and that have at least one L/C misregulated gene in *cli186 *in the supernode include "glycolysis/gluconeogenesis", "nitrogen metabolism", "carbon fixation", "expressed protein", "pyruvate metabolism", "protein kinase", nicotinate and nicotinamide metabolism" and "phenylalanine metabolism" (Figure 2, yellow nodes). Of the 108 processes shown as supernodes in the wild type supernode network, 41 of them are perturbed by the misregulation of one or more genes in *cli186*. Thus, genes that are misregulated in *cli186 *are functionally associated.

A supernode network was also created using the 216 misregulated genes in *cli186 *to visualize the processes that contain the largest number of genes that are misregulated in *cli186*, to see the number of connections and with which other nodes they are connected [see Additional file 6]. These supernodes, representing biological processes, indicate that the regulation of genes in these processes are perturbed in *cli186*. The supernodes with the highest number of genes misregulated by L/C in *cli186 *include, "nitrogen metabolism", "glycolysis/gluconeogenesis", "carbon fixation" and "expressed protein". Prominently, within this supernode network of *cli186 *L/C misregulated genes is a homeobox leucine zipper protein connected via protein-DNA connections to the supernodes that represent metabolic processes such as "nitrogen metabolism", "glycolysis/gluconeogenesis" and "carbon fixation" among others. Within this supernode network there are additional proteins with DNA binding and/or transcriptional activity but do not exhibit the striking connectivity of the homeobox leucine zipper protein [see Additional file 6].

### A homeobox-leucine zipper transcription factor (*HAT22*) integrates metabolic networks

**Figure 3:**
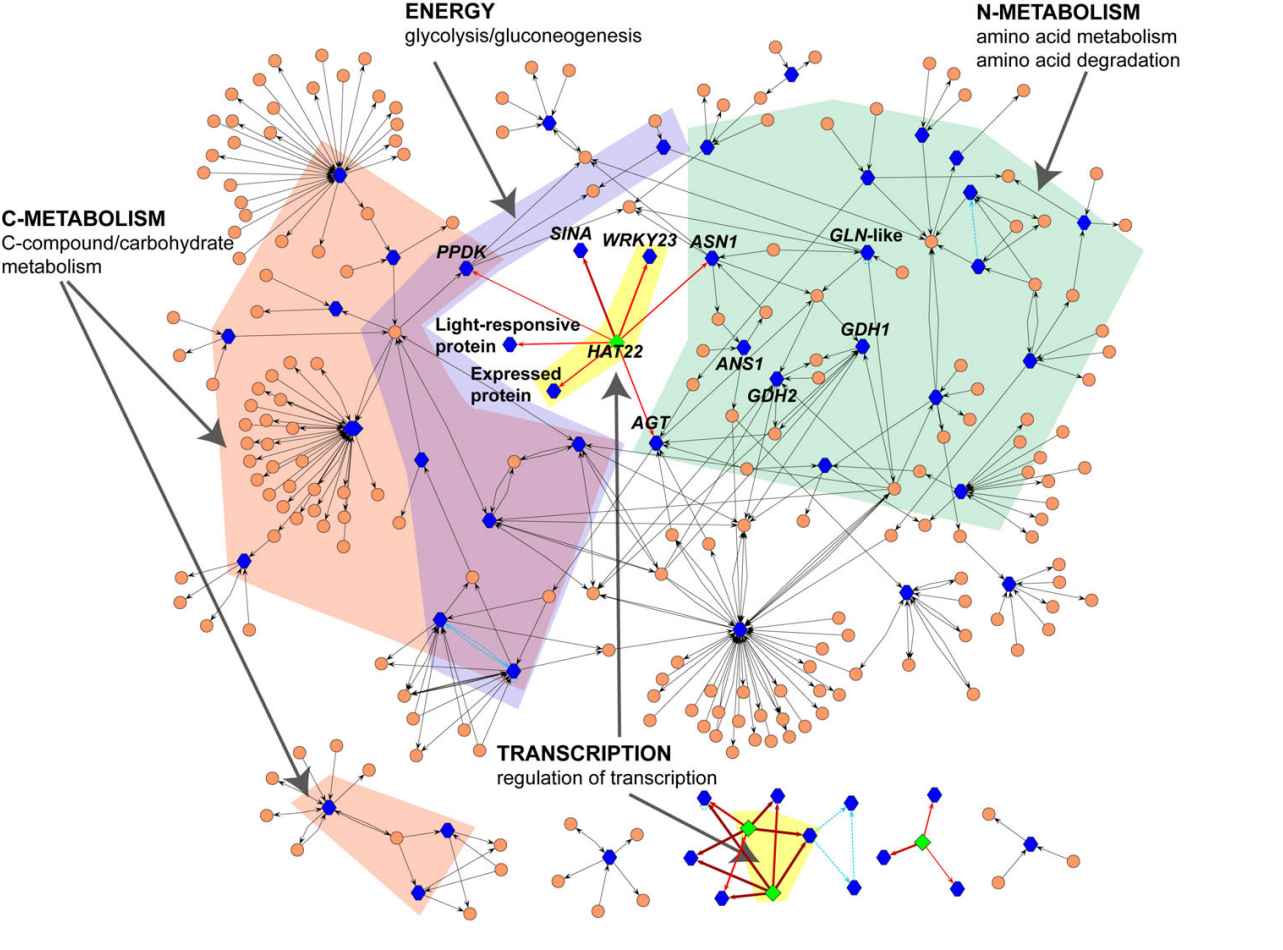
A metabolic and regulatory sub-network created from the 216 misregulated genes in *cli186*. This is a sub-network of the 216 misregulated genes that was extracted from the larger multinetwork created using the 966 L/C regulated genes in WT [see Additional file 5] and visualized using Cytoscape [54]. Nodes representing genes (blue hexagons), genes annotated to be transcription factors (green diamonds) or metabolites (peach circles) are connected via edges. The type of edge indicates if the interaction is metabolic (grey arrows), protein-DNA regulation (red arrows) or protein-protein (blue dashed lines). Protein-DNA interactions are supported by the presence of one or more binding sites within the promoter of that gene for that particular transcription factor (see Methods). This is a connected network comprised of 60/216 misregulated genes in *cli186 *in which all of the nodes represented are misregulated in *cli186*.

To gain a gene-by-gene network view of the connectivity of the 216 L/C misregulated genes in *cli186*, the 216 genes were used to directly query the Arabidopsis multinetwork (Figure 3) [see Additional file 7]. A number of interactions are visualized within this sub-network including: (1) metabolic networks (2) protein:protein interaction networks and (3) protein:DNA regulatory interaction networks [21] (see Methods). Nodes representing genes (blue hexagons), genes annotated to be transcription factors (green diamonds) or metabolites (peach circles) are connected via edges. The type of edge indicates if the interaction is metabolic (black arrows), protein:DNA regulation (red arrows) or protein-protein interactions (blue dashed lines). Predicted transcription factor target gene edge connections are based on interactions available from AGRIS [39], the interolog and regulogs described previously [40] and the statistical over-representation of the transcription factor binding sites within the target gene promoter and the correlation (p < 0.01 and r > 0.5) of expression of the transcription factor and target gene across all 16 experiments (see Methods). This analysis shows one large network comprised of 60/216 genes, all of which are L/C misregulated in *cli186*.

The gene network analysis reveals that a number of enzymatically and physically connected genes involved in the biological processes "C-compound/carbohydrate metabolism", "amino acid metabolism" and glycolysis/gluconeogenesis" are misregulated by L/C in *cli186*. Thus, these metabolic processes are likely to be overall perturbed in *cli186*. Moreover, at least one gene in these three 'supernodes' has a regulatory edge connected to *HAT22*, a homeobox-leucine zipper protein 22. The genes that contain regulatory protein:DNA edge connections to *HAT22 *include *ASN1 *(asparagine synthetase) (the gene used in the genetic screen to isolate the *cli186 *mutant), *PPDK *(pyruvate phosphate dikinase) and *AGT *(alanine glyoxylate aminotransferase). This network result suggests that *HAT22 *may be involved in coordinating the regulation of the target genes in the three related metabolic processes "amino acid metabolism", "C-compound/carbohydrate metabolism" and "glycolysis/gluconeogenesis" (Figure 3) [see Additional file 7]. Furthermore, *HAT22 *shows additional regulatory edge connections to four other genes, three of which are known regulatory genes (*WRKY23 *(At2g47260), *SINA *(At3g61790), a light-regulated signaling protein (At3g26740)) and one is an unknown gene annotated as an expressed protein (At3g20340). *WRKY23 *is one member of the family of *WRKY *transcription factors, *SINA *(seven in absentia protein family) is a gene required for R7 photoreceptor development in Drosophila [41] but whose function is currently unknown in Arabidopsis, the light responsive protein is involved in signaling and the 'expressed protein' is annotated as having a gene regulatory function. Interestingly, *ASN1*, the gene used to construct the transgenic plants used in the selection of the *cli *mutants, is included in this *cli186 *gene network and is connected to the *HAT22 *hub. Strikingly, there are a large number of genes in this network annotated to "amino acid metabolism" and "amino acid degradation", all of which are misregulated in *cli186*. Other genes within this metabolic subnetwork are genes related to or are byproducts of Asn synthesis and degradation and include the following: At3g47340 (*ASN1*, asparagine synthetase), At3g16150 (*ANS1*, asparaginase), At5g18170 (*GDH1*, glutamate dehydrogenase 1), At5g07440 (*GDH2*, glutamate dehydrogenase 2) and At1g55090 (glutamine-dependent NAD (+) synthetase). The misregulation of six of seven HAT22 target genes in the network have been validated via q-PCR and all of these genes (*SINA*, At3g61790; *WRKY23*, At2g47260; *ASN1*, At3g47340; *PPDK*, At4g15530; light-regulated protein, At3g26740; *AGT*, At3g08860), including HAT22 itself show misregulation in *cli186 *when compared to WT [see Additional file 8].

### Integration of *cli186 *with light signaling mutants

As the genome scale analysis suggests that *cli186 *is specifically impaired in the regulation of genes affected by L/C interactions, it was of interest to compare gene regulation in *cli186 *to other Arabidopsis mutants impaired in light sensing/signaling. We compared morphological and molecular phenotypes where applicable, to gain a preliminary idea of where CLI186 functions with respect to known light signaling mutants. *Cli186 *exhibits shorter hypocotyls and opened cotyledons during etiolated growth, similar to other known constitutively photomorphogenic mutants. Comparison of the molecular phenotypes of *cli186 *with *cop *[42] and *det *[43] mutants reveals that unlike *cop *and *det *mutants, *cli186 *does not exhibit induced expression of the *RBCL *and *CAB *genes in etiolated plants. Furthermore, based on microarray analysis, the *cli186 *mutant displayed a loss of light regulation rather than a constitutive light response in the dark based on microarray analysis. As this is more consistent with a mutation in the phytochrome and/or cryptochrome signaling pathways, the growth phenotype of *cli186 *was analyzed alongside the photoreceptor mutants, *phyA-201 *[44]*phyB-5 *[45] and *cry1-304/cry2 *[46] in various light conditions (e.g. continuous far-red light for *phyA-201*, continuous red light for *phyB-5 *and continuous blue light for *cry1-304*/*cry2-1*). *Cli186 *exhibited an inhibition in hypocotyl elongation similar to wild-type under all light conditions, whereas the photoreceptor mutants displayed elongated hypocotyls compared to their wild type. These preliminary studies indicate that *cli186 *is not an allele of either the *phyA*, *phyB*, *cry1 *or *cry2 *mutants (data not shown).

Since it was determined that *cli186 *is not in an upstream component of light signaling (*cop*, *det, phyA*, *phyB*, *cry1 *or *cry2 *mutant), we attempted to place *CLI186 *as a downstream component in the context of other known light or carbon signaling pathways by comparing available microarray data from light signaling mutants with microarray data from *cli186*. We focused on the *phyA *and *phyB *microarray studies that were the most similar with respect to growth conditions [47,48] used for the *cli186 *studies described herein. A comparison of genes misregulated by *phyA *and/or *phyB *[47,48] with the 216 L/C misregulated genes in *cli186 *reveals sixty-eight genes that are shared among the misregulated genes in *cli186 *and the *phy *data sets. As PHYA is the predominant phytochrome type in etiolated seedlings, and because the *ASN1 *misregulated molecular phenotype of *cli186 *is more pronounced in etiolated seedlings, CLI186 may function as a downstream component of the PHYA pathway that integrates L and C signaling pathways. The involvement of CLI186 in a PHYA or PHYB pathway does not preclude it from also potentially functioning downstream of any of the other photoreceptors. Thus, it will be beneficial to investigate *cli186 *in the context of other light and carbon signaling mutants when microarray data becomes available.

## Discussion

### *CLI186*: An integrator of light and carbon signaling interactions

**Figure 4:**
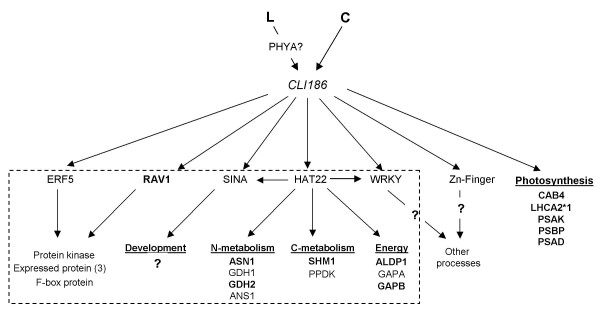
A proposed model: *CLI186 *is an integrator of L and C signaling interactions. L and C signaling interactions converge via *CLI186 *that may, in turn, act on a number of downstream regulatory factors such as HAT22, SINA, RAV1, ERF5, WRKY and a Zn-finger protein. These regulatory proteins may regulate a number of genes involved in various biological processes such as N-metabolism, C-metabolism and Energy among others. Genes involved in the light reactions of photosynthesis (CAB4, LHCA2*1, etc.) were not represented within the multinetwork and so it remains unclear how *CLI186 *may target these genes for regulation. Genes in bold are those that exhibit misregulation in the *cli186 *mutant and in *phyA *and *phyB *mutants, supporting the hypothesis that the light signal integrated with carbon may be perceived through a phytochrome pathway. The genes within the dashed box indicate a relationship supported by the network analyses. All genes depicted within this hierarchical network are misregulated in the *cli186 *mutant, with the exception of *PHYA*. This molecular hierarchy depicted is supported by our analysis and interpretation of the microarray analyses carried out for *cli186*.

The systems wide studies herein suggest that the *cli186 *mutation blocks the integration of light and carbon signaling pathways that occur in wild-type Arabidopsis. Furthermore, comparative analysis of microarray data from *cli186 *and *phy *mutants was used to derive the preliminary hypothesis that *CLI186 *functions downstream of a light signaling pathway, that is, at least in part, mediated by phytochrome (possibly phyA) and a carbon signaling pathway, mediated by a yet undetermined sensor or modulator (Figure 4). Our analysis of gene networks that are misregulated in *cli186*, combined with a comparative analysis of genes misregulated in *phyA *and *phyB *mutants enabled us to place *CLI186 *in a molecular hierarchy, depicted in Figure 4. Our hypothesis that *CLI186 *acts downstream of phyA is also based on the dramatic changes in L/C regulated gene expression observed in etiolated plants. While the misregulation by light and carbon (LC) of *ASN1 *expression in light-grown *cli186 *seedlings was modest (Figure 1c), it was sufficient to select *cli *mutants, based on misregulation of the *ASN1*::*HPT2 *construct. Since the *cli186 *mutant exhibits delayed greening and problems with seed set (data not shown), the notion that CLI186 also plays a role in light-grown seedling development is currently being investigated.

Among the 216 genes misregulated by L/C in *cli186*, nine genes are transcription factors or genes with regulatory function, of which six are present in the large L/C multinetwork (*HAT22*, *RAV1*, *ERF5*, *WRKY*, *SINA *and one unnamed C_3_HC_4_-Zn finger binding protein). HAT22, a homeodomain leucine zipper protein is of particular interest, as it appears to be a network hub that is predicated to target the promoters of 53 genes in the multinetwork [see Additional file 5]. Seven of these 53 target genes of *HAT22 *are also misregulated in *cli186 *and have been validated via qPCR in their misregulation [see Additional file 8]. The misregulation of *HAT22 *in *cli186 *suggests that *HAT22 *is a putative transcription factor hub that functions downstream of *CLI186 *to integrate L/C regulation of genes involved in metabolic networks related to C-metabolism, N-metabolism and Energy, as well as regulatory networks involved in transcription. A number of the genes in these *HAT22 *target metabolic networks (e.g. *ASN1*, *ANS*, *GDH2*, *SHM1*, *ALDP1 *and *GAPB*) are also affected in *phyA *or *phyB *[47,48] mutants, supporting the gene network hierarchy of *CLI186 *being involved in the integration of L and C signaling as shown in the model in Figure 4. It is noteworthy that MIPS funcat analysis reveals that genes involved in photosynthesis are also over-represented in the list of misregulated genes in *cli186 *(Table 2) and that a number of these genes are also misregulated in the *phyA *and *phyB *mutants. The genes involved in the light reactions of photosynthesis are not in the current version of the Arabidopsis multinetwork [21] because at present, there are no edges connecting these photosynthesis genes to the rest of the network (e.g. via protein:protein interactions). This could be because the inferred protein:protein interactions in the current Arabidopsis multinetwork are based on experimentally proven protein:protein interactions in worm, fly or yeast [49]. Thus, a number of genes and/or interactions specific to plants, such as those involved in photosynthesis are not yet represented in the Arabidopsis multinetwork. The other five transcription factors, acting downstream of *CLI186*, may regulate genes involved in photosynthesis or other metabolic and regulatory networks that are not represented in the multinetwork or that are not present in the dataset used for the analyses in this report. These transcription factors and their role in the L/C regulation of genes are currently under investigation. The model presented in Figure 4 is one hypothesized manner in which CLI186 may act to integrate L and C signaling interactions that best accounts for and fits our analysis and interpretation of the microarray analyses carried out for *cli186*.

This systems based analysis of the *cli186 *mutant suggests the existence of a master regulator of L/C signaling interactions. Furthermore, our network analysis defines HAT22 as a regulatory hub that plays a major role in integrating the L and C regulation of genes in the biological processes, "amino acid metabolism" and "glycolysis and gluconeogenesis". Thus, *HAT22 *could hypothetically play the role of *CLI186 *as a master regulator however, *HAT22 *does not encode the affected gene in *cli186 *based on (1) the presence of *HAT22 *mRNA in the *cli186 *mutant (data not shown); (2) the fact that *HAT22 *passes the first criteria for filtering of microarray data (*i.e. *probes must be called 'present' in baseline or treatment hybridizations) and (3) location of *HAT22 *in the genome (chr 4). Although, *HAT22 *is ruled out as a *cli186 *candidate, other transcription factors, miRNAs or genes with regulatory roles such as those involved in chromatin remodeling, that are not within the scope of this analysis are possible *cli186 *candidates and are currently under investigation. Interestingly, of the 216 misregulated genes in *cli186*, almost 80% of them exhibit a dampening of regulation (either induction or repression) via the interaction of L and C signaling pathways, suggesting that *CLI186 *may be positive regulatory factor.

## Conclusion

In this study we have taken an integrated genetic, genomic and systems approach to define gene networks that are regulated by the interaction of light and carbon signaling and that are perturbed in *cli186*. Using this approach, we have developed the biological hypothesis that CLI186 is a regulatory hub affecting the integration of L and C signaling and exerts its effects via downstream transcription factors which in turn regulate genes involved in essential biological processes such as amino acid metabolism and glycolysis/gluconeogenesis. More common methods used thus far for genome-wide studies in Arabidopsis such as cataloging genes based on biological processes and providing gene lists, would not have enabled us to gain a systems biology understanding of how CLI186 may act to integrate L and C signaling interactions via HAT22 and other downstream transcription factors. We believe that in addition to elucidating the factors involved in the integration of L and C signaling pathways, that this work may serve as a case study wherein the analysis of genomic data in a systems/network context can help define the role of a mutated gene. Moreover, this type of analysis could potentially be used to determine which mutants in a genetic screen are potential network hubs and should be targeted for gene cloning efforts.

## Methods

### Construction of *ASN1::HPT2 *reporter gene transgenic line

Transgenic *Arabidopsis thaliana *plants were constructed in which the genome contained the 5' upstream region of *ASN1 *from -148 to +120 fused in a transcriptional fusion to the hygromycin phosphotransferase (*HPT2*) gene and to a *GUS *gene [30]. This 148-bp region of the *ASN1 *promoter has previously been shown to include the regulatory *cis*-elements necessary for repression of the gene in response to light [30,31] and carbon [31]. This *ASN1-HPT2*/*ASN1-GUS *construct was further subcloned into the Ti binary vector PBI101.1, introduced into *Agrobacterium tumefaciens *LBA4404 and used to transform the Columbia ecotype of Arabidopsis via vacuum infiltration [31]. Among the kanamycin-resistant independent transformants, only plants containing a single insertion were selected for further characterization. The transgenic line exhibiting the most dramatic level of light and sucrose repression of GUS expression was chosen for a genetic screen to select for plants defective in light and/or carbon signaling [31].

### Screening for *cli *mutants

An unmutagenized (wild-type) line harboring the *ASN1-HPT2 *transgene was mutagenized with fast-neutron irradiation or ethylmethane sulfonate (EMS). 20,000 M2 seeds from 763 individual M1 fast-neutron irradiated lines and 23,000 M2 seeds from 579 M1 EMS-generated lines were screened for resistance to hygromycin when grown in the presence of light and sucrose. Mutagenized seed and controls were surface-sterilized, plated on designated media and stratified for 48 hours at 8°C. Seeds were grown on media containing 1× Basal MS (Life Technologies, Long Island, NY), 0.9% (w/v) bactoagar, pH adjusted to 5.7 with KOH, supplemented with 2 mM KNO_3_, 0.5% sucrose and 15 μg/ml hygromycin (Gibco-BRL). Controls consisted of unmutagenized seeds containing the *ASN1-HPT2 *transgene and a transgenic line containing the *HPT *transgene driven by a NOS promoter, allowing for constitutive expression of the *HPT2 *gene. Two fast-neutron generated and 25 EMS generated hygromycin-resistant mutants were isolated based on their growth phenotype when compared to the unmutagenized *ASN1::HPT2 *containing line: hygromcyin-resistant plants exhibited longer root length and green primary and secondary leaves. Mutants demonstrating a consistent, heritable hygromycin-resistant phenotype were characterized further using quantitative PCR to determine aberrant expression of the endogenous *ASN1 *gene.

### Plant Growth and RNA Isolation

Wild-type and mutant seeds were grown and RNA was isolated as described in a previous study [17]. Seeds stocks for *phyA-201 *and *phyB-5 *were obtained from the Arabidopsis Biological Resource Center (Ohio State University) and *cry1-304/cry2-1 *from Dr. Chentao Lin (University of California, Los Angeles).

### Quantitative PCR

RNA was isolated from whole plants using a phenol extraction protocol as previously described [17]. The RNA samples used for quantitative PCR are the same as those used for the microarray analysis in this study. cDNA synthesis using 1.0 μg total RNA was carried out according to Invitrogen (catalog no. 11146-024). Subsequent real-time quantitative PCR was carried out with a LightCycler (Roche Diagnostics, Mannheim, Germany). PCR amplification in a 20 μl reaction volume consisted of a master mixture containing DNA Taq polymerase, dNTP mixture and buffer (LightCycler DNA Master SYBR Green 1, Roche Diagnostics, catalog no. 2158817), 4 mM MgCl_2_, 0.9 μM of each primer and cDNA in a glass capillary tube. Primers spanned at least one intron for each gene analyzed and were designed using the LightCycler probe design software (Roche). The primers were synthesized at Invitrogen Life Technologies (Carlsbad, CA). Quantitative PCR using hybridization probes was carried out as previously described for *ASN1 *and At4g24550, a putative clathrin coat assembly protein [17]. The following primers were used for amplification: HAT22 (At4g37790) 5' GCAGACCTCGTCCCAC 3'(forward primer), 5' GCACATAGTCAAAGTCGC 3' (reverse primer); SINA (At3g61790) 5'CACTGCTTCGGTCAAT 3' (forward primer), 5' AGGCCAAATCGGTGAG 3' (reverse primer); WRKY23 (At2g47260) 5' TGGTTATCGCTGGCGA 3' (forward primer), 5'AGCGTGGCTATTAAGGT 3' (reverse primer); PPDK (At4g15530) 5' TGCAGGCTCGGAATAT 3'(forward primer), 5' AGGGTCGTGCTGTAAGA 3' (reverse primer); AGT (At3g08860) 5' ATCGTAGAGCTTGCTCC 3' (forward primer), 5' CCAACGAGGTTAGCATT 3' (reverse primer); Light-regulated protein (At3g26740) 5' GCTCTGTTTATCAAACCAACT 3' (forward primer), 5' CCAAGATCATCGCAGGC 3' (reverse primer). Thermal cycling was performed as follows: initial denaturation at 95°C for 2 min, followed by 45 cycles of denaturation at 95°C for 0 s, annealing at 55°C for 5 s (*HAT22, SINA *and *PPDK*) or 60°C for 10 s (*WRKY23, AGT *and light-regulated protein) and extension at 72°C for 20 s. Melting curve analyses were carried out for all amplification reactions as follows: one cycle of an initial denaturation at 95°C for 0 s, annealing at 50°C for 5 s and another denaturation at 95°C for 0 s with a slope of 0.1°C/s. Standards were prepared with a 10-fold serial dilution (10^-4 ^to 10 pg) of the PCR products and were run under the same PCR conditions used for the samples. The absolute amount of mRNA [ng/ml] for all samples was corrected/normalized according to the amount of At4g24550, a putative clathrin coat assembly protein.

### Microarray Analysis and Data Filtering

Preparation of cDNA and cRNA for microarray analysis was carried out as previously described [18]. 15 μg of cRNA was used for hybridization (16 hours at 42°C) to the Arabidopsis Genome ATH1 array (Affymetrix). Washing, staining, and scanning were performed as recommended by the Affymetrix instruction manual. Expression analysis was performed with the Affymetrix Microarray Suite software (version 5.0) set at default values with a target intensity set to 150. Two biological replicates for each treatment were carried out.

Wild-type and *cli186 *plants were subjected to four different light and/or carbon treatment conditions (-L+C; +L-C; +L+C; -L-C). All experiments for Q-PCR were carried out independently in triplicate where two of these experiments were used for microarray analysis. Treatment with no light and no carbon for either wild-type or *cli186*, (-L-C) served as the control treatment and was used as the background to which all other treatments were compared (+L-C; +L+C; -L+C). Each of two background treatments (*e.g. *-C-L) were compared with both treatment replicates (e.g. -L+C) providing a stringent four-way comparison. All treatments were started at 9 a.m. to control for possible circadian fluctuations. Data analysis from wild-type and *cli186 *arrays was carried out simultaneously to classify genes based on their regulation by light and/or carbon across all 24 comparisons (4 comparisons/treatment × 3 treatments × 2 genotypes). Genes were retained for the analysis of wild-type and *cli186 *if the values in all treatments met the following criteria. Filter 1: the detection calls must not be called absent (A) or marginal (M) in both background and treatment conditions. On average, this filter removed 10,450 genes/comparison, leaving approximately 12,360 genes/comparison. Filter 2: if a gene is called absent (A) or marginal (M) in one hybridization (control or treatment) then the signal in the present (P) call must be > or = 50. On average, this filter removed 784 genes/comparison, leaving approximately 10,576 genes/comparison. Filter 3: the difference calls must be the same in 3 out of 4 comparisons. Application of these three filters to both wild-type and *cli186 *resulted in a reliable dataset. Using the filtered dataset of 1,625 genes, patterns of regulation across different treatments were assigned to each gene using the Affymetrix difference calls.

### 'Metric' Classification System

If a gene was found differentially regulated in two or more conditions using the Affymetrix software, an ANOVA analysis and Tukey's post-hoc analysis was carried out to rank the mean expression in the treatments in wild-type and *cli186*. Multiple testing was addressed by controlling the false discovery rate (FDR) at 5% as described previously [34]. If the mean of the signal log ratios of a gene were significantly different in wild-type versus *cli186 *in one treatment, that gene was numerically ranked based on the level and direction of change. For example, if a gene exhibits significantly higher expression in wild-type than in *cli186 *in a particular treatment, expression for that gene may be summarized with a numerical rank of 'two' in wild-type and 'one' in *cli186*. Negative numerical rankings indicate repression of gene expression. Differences in numbers in wild-type versus *cli186 *in any one treatment indicate a statistically significant difference in gene expression between the two genotypes.

Examples of how genes are classified according to their regulation in wild-type and misregulation in *cli186 *are shown as additional data [see Additional file 9]. Values of "0" signify no change, while induction is positive and repression is a negative number. The treatments are ordered L, LC, and C [see Additional file 9]. As an example of this system, using Tukey's post-hoc analysis for a pair-wise comparison of all treatments between wild-type and *cli186*, significant differences in expression of Gene A were observed in the L-only treatment, thus Gene A shows the numerical assignment, "2 1 1" for wild-type, and "1 1 1" for *cli186*. Therefore, genes that are assigned the numerical values of "2 1 1"/"1 1 1" (L LC C) are determined to be misregulated in *cli186 *only in L-only treatments. The numbers assigned to each of the genes also indicate the direction of regulation observed in any one of the treatments. In a second example, the numerical assignment for Gene B, "-1 -1 -1" (L LC C) for wild-type and "-1 -1 -2" (L LC C) for *cli1186 *indicates that Gene B is deduced to be mis-regulated in *cli186 *only in the C-only treatment [see Additional file 9]. Further examples of how patterns of gene expression may be classified and used to identify misregulated genes in *cli186 *are in Additional data (Genes C-G) [see Additional file 9]. In total, the filtered dataset of 1,625 genes exhibited 46 different patterns of L, CL and C regulation. These combinations of C and L regulation were compared between wild-type and *cli186*, and used to classify genes according to their misregulation in *cli186 *in any one of or combination of the treatment conditions.

### Three-Way ANOVA

To look at the genes regulated by either C and/or L in wild-type and the *cli *mutant a 3-way ANOVA analysis with three dichotomous categorical variables (Carbon, Light, and Genotype) was performed. The p-values were corrected for multiple testing using a FDR method with a cutoff of 0.05 or 5% FDR. Our results indicate that the majority of the regulated and misregulated genes determined using a 3-way ANOVA analysis is very similar to those obtained from the method discussed above [see Additional file 4]. The model used is as follows:

Gene expression ~carbon + light + carbon:light + carbon:genotype + light:genotype + carbon:light:genotype -1

Our model assumes no intercept and thus contains a minus 1.

### Arabidopsis Multinetwork

The Arabidopsis multinetwork consists of genes (nodes) connected by edges that represent interactions based on: (1) metabolic pathways (2) protein-protein interactions and (3) protein-DNA regulatory interactions. Support for the gene connections in metabolic pathways are based on information from KEGG [50], and AraCyc [51] databases. Support for protein-protein interactions and protein-DNA interactions are based on information from DIP [49], BIND [52] and Transfac [53]. Predicted protein-DNA interactions are supported by those available from AGRIS [39], the interolog and regulogs [40] and a method developed in our lab used to predict protein-DNA interactions (see 'Predicting Protein:DNA Interactions' below). At present, this Arabidopsis multinetwork contains 6,176 gene nodes, 1,459 metabolite nodes and 230,900 interactions "edges" connecting the nodes. Two nodes can have multiple edge connections. Querying this multinetwork with a gene list (e.g. from a microarray experiment) and visualization using the Cytoscape software [54] displays all connections between the genes of interest and other biological molecules.

### Predicting Protein:DNA Interactions

Protein:DNA interactions were generated using biologically responsive elements taken from the AGRIS database [39] to scan upstream sequence using the DNA pattern search tool available from RSA tools [55]. 1000 bp of the upstream sequence of all genes were scanned in the forward and reverse directions without overlap and matches to the consensus sequence were reported based on their frequency within the upstream region (starting from -1000 bp upstream). Those binding sites having a frequency greater then a set Z-score (average + 2 standard deviations) were considered over-represented and therefore significant based on our own analysis of frequency effects within the genome as well as previous work in eukaryotic species showing a correlation between increased frequency of a binding site and a change in expression [56].

### Statistics for Multinetwork

It was of interest to determine the significance of the multinetwork created from the 966 L/C regulated genes and the 216 misregulated genes. The significance of the presence of the genes in the network was determined as well as the connectivity among nodes (genes) in the network.

The significance of the presence of the 966 L/C regulated genes within the Arabidopsis Multinetwork was determined by calculating how many nodes from the 966 L/C regulated dataset (dataset Reg) are present in the Multinetwork (dataset M) and then asking, if a random sample size of 966 genes were sampled 10,000 times from the entire Arabidopsis genome of 22,811 nodes, how many times could we expect to find the same number of nodes present in the Multinetwork. It was found that there are 542 nodes (dataset Reg_M) from the 966 L/C regulated dataset that are present in the Multinetwork and in no cases (out of 10,000) does an arbitrary set of 966 nodes out of 22,811 intersect with such a large cardinality with those present in the Multinetwork (p value < 10^-4^). We also determined the significance of the connectivity of the multinetwork that was created from the 966 L/C regulated dataset. Here, the number of the genes (nodes) that are shared among dataset Reg_M were determined and of those 542 genes (dataset Reg_M) that are shared among the two datasets, the number of edges were determined, of which there are 3356. It was then asked, if a random sample size of 542 or more genes was sampled 10,000 times from the Multinetwork, how many times could we expect to find 542 genes that have 3356 edges. In no cases, out of 10,000, did a sample size of 542 genes or more out of the Multinetwork contain 3356 edges (p value = 0.0000).

The significance of the 216 misregulated genes (dataset Mis) within the multinetwork that was created by using the 966 L/C misregulated genes was determined. Here, the intersection between the genes in dataset Reg_M and the 216 misregulated genes (dataset Mis) was determined to be 92 genes. It was then asked, if a random sample size of 216 genes was sampled 10,000 times from the Arabidopsis genome, how many times could we expect to find 92 genes that are in dataset Reg_M. In no cases (out of 10,000) does an arbitrary set of 216 nodes out of 22,811 intersect with such a large cardinality with those present in dataset Reg_M (p value < 10^-4^). The significance of the connectivity of the 216 L/C misregulated genes in the Multinetwork was investigated by determining how many edges are induced in dataset M from the genes in database Mis (i.e. an edge (g1, g2) is induced if both g1 and g2 are in Mis). The answer is 289. We then asked how many times can one expect to find 298 edges induced in M from a randomly selected group of 216 genes from dataset Reg_M. In 418 cases, out of 10,000, did a sample size of 216 genes from dataset Reg_M induce 298 edges in M (p value = 0.0418).

### Microarray Data

The microarray data for this article have been deposited in the ArrayExpress database, accession number E-MEXP-1112.

## Authors' contributions

KET and MJS carried out the molecular genetic studies and microarray analysis and were involved in the experimental design. KET was involved in analysis of the multinetworks. RAG was involved in data analysis, contributed new tools for analysis and helped design the research. IM carried out the q-PCR validation of expression of HAT22 target genes. MSK participated in the 3-way ANOVA analysis. MSK and DN participated in developing and updating the new tools for the multinetwork analyses carried out herein. DES carried out the statistical analysis for the multinetworks. GMC participated in the design and coordination of the study. KET, MJS and GMC wrote the manscript. All authors have read and approved the manuscript.

## Supplementary Material

Additional file 1 Metric classes and the genes within each class. A list of 1,625 filtered genes and their 'metric' classification for L/C regulated genes in wild-type and *cli186*. Click here for file

Additional file 2 Genes L/C regulated in wild-type and misregulated in *cli186*. List of genes and gene annotations classified as being 'regulated' in wild-type and *cli186 *or 'misregulated' in *cli186 *according to 'metric' classification. Click here for file

Additional file 3 Patterns of L/C misregulation in *cli186*. Table showing patterns of misregulation observed among the 216 misregulated genes in *cli186*. Click here for file

Additional file 4 ANOVA analysis. Lists of regulated and misregulated genes as determined via 3-way ANOVA analysis. Click here for file

Additional file 5 Multinetwork of L/C regulated genes in wild-type. Metabolic and regulatory network created using the Arabidopsis Multinetwork Tool [21], using the 966 L/C regulated set of genes and visualized with Cytoscape [54]. Click here for file

Additional file 6 Supernode network of misregulated genes in *cli186*. Supernode network created from the 216 misregulated genes in *cli186*. Click here for file

Additional file 7 Misregulated multinetwork in *cli186*. Metabolic and regulatory network created using the Arabidopsis Multinetwork Tool [21], using the 216 L/C misregulated set of genes from *cli186 *and visualized with Cytoscape [54]. Click here for file

Additional file 8 Metric classification. Examples of 'metric classification' system designed to determine L. LC, C misregulated genes in *cli186*. Click here for file

Additional file 9 Q-PCR validation. q-PCR validation of misregulation of HAT22 and its target genes. Click here for file

## References

[B1] Fankhauser C, Chory J (1997). Light control of plant development. Annu Rev Plant Physiol Plant Mol Biol.

[B2] Casal JJ, Yanovsky MJ (2005). Regulation of gene expression by light. Int J Dev Biol.

[B3] Neff MM, Fankhauser C, Chory J (2000). Light: An indicator of time and place. Genes Dev.

[B4] Moller SG, Ingles PJ, Whitelam GC (2002). The cell biology of phytochrome signaling. New Physiol.

[B5] Nagy F, Schafer E (2002). Phytochromes control photomorphogenesis by differentially regulated, interacting signaling pathways in higher plants. Annu Rev Plant Physiol Plant Mol Biol.

[B6] Franklin KA, Larner VS, Whitelam GC (2005). The signal transducing photoreceptors of plants. Int J Dev Biol.

[B7] Koch KE (1996). Carbohydrate-modulated gene expression in plants. Annu Rev Plant Physiol Plant Mol Biol.

[B8] Rolland F, Moore B, Sheen J (2002). Sugar sensing and signaling in plants. Plant Cell Sup.

[B9] Rolland F, Baena-Gonzalez E, Sheen J (2006). Sugar sensing and signaling in plants: conserved and novel mechanisms. Annu Rev Plant Biol.

[B10] Rolland F, Sheen J (2005). Sugar sensing and signaling networks in plants. Biochemical Soc Trans.

[B11] Gibson S (2005). Control of plant development and gene expression by sugar signaling. Cur Opinion Plant Sci.

[B12] Osuna D, Usadel B, Morcuende R, Gibon Y, Blasing O, Hohne M, Gunter M, Kamlage B, Trethewey R, Scheible W-R, Stitt M (2007). Temporal responses of transcripts, enzyme activities and metabolites after adding sucrose to carbon-deprived Arabidopsis seedlings. Plant J.

[B13] Moore B, Zhou L, Rolland F, Hall Q, Cheng W-H, Liu Y-X, Hwang I, Jones T, Sheen J (2003). Role of the Arabidopsis glucose sensor HXK1 in nutrient, light and hormonal signaling. Science.

[B14] Barnes SA, Nishizawa NK, Quaggio RB, Whitelam GC, Chua NH (1996). Far-red light blocks greening of Arabidopsis seedlings via a phytochrome A-mediated change in plastid development. Plant Cell.

[B15] Dijkwel PP, Huijser C, Weisbeek PJ, Chua NH, Smeekens S (1997). Sucrose control of phytochrome A signaling in Arabidopsis. Plant Cell.

[B16] Short T (1999). Over-expression of Arabidopsis phytochrome B inhibits phytochrome A function in the presence of sucrose. Plant Physiol.

[B17] Thum KE, Shasha DE, Lejay LV, Coruzzi GM (2003). Light and carbon signaling pathways. Modeling circuits of interactions. Plant Physiol.

[B18] Thum KE, Shin MJ, Palenchar PM, Kouranov A, Coruzzi GM (2004). Genome-wide investigation of light and carbon signaling interactions in Arabidopsis. Genome Biol.

[B19] Palenchar P, Kouranov A, Lejay L, Coruzzi GM (2004). Genome-wide patterns of carbon and nitrogen regulation of gene expression validate the combined carbon and nitrogen (CN)-signaling hypothesis in plants. Genome Biol.

[B20] Price J, Laxmi A, St. Martin SK, Jang JC (2004). Global transcription profiling reveals multiple sugar signal transduction mechanisms in Arabidopsis. Plant Cell.

[B21] Gutierrez RA, Lejay LV, Dean A, Chiaromonte F, Shasha DE, Coruzzi GM (2007). Qualitative network models and genome-wide expression data define carbon/nitrogen-responsive molecular machines in Arabidopsis. Genome Biol.

[B22] Li Y, Lee KK, Walsh S, Smith C, Hadingham S, Sorefan K, Cawley G, Bevan MW (2006). Establishing glucose- and ABA-regulated transcription networks in Arabidopsis by microarray analysis and promoter classification using a relevance vector machine. Genome Res.

[B23] Blasing OE, Gibon Y, Gunther M, Hohne M, Morcuende R, Osuna D, Thimm O, Usadel B, Scheible W-R, Stitt M (2005). Sugars and circadian regulation make major contributions to the global regulation of diurnal gene expression in Arabidopsis. Plant Cell.

[B24] Lam HM, Hsieh MH, Coruzzi GM (1998). Reciprocal regulation of distinct asparagine synthetase genes by light and metabolites in *Arabidopsis thaliana*. Plant J.

[B25] Oliveira IC, Coruzzi GM (1999). Carbon and amino acids reciprocally modulate the expression of glutamine synthetase in Arabidopsis. Plant Physiol.

[B26] Rook F, Bevan MW (2003). Genetic approaches to understanding sugar-response pathways. Journal of Exp Bot.

[B27] Laby RJ, Kincaid MS, Kim D, Gibson SI (2000). The Arabidopsis sugar-insensitive mutants sis4 and sis5 are defective in abscisic acid synthesis and response. Plant J.

[B28] Huijser C, Kortstee A, Pego J, Weisbeek P, Wisman E, Smeekens S (2000). The Arabidopsis SUCROSE-UNCOUPLED-6 gene is identical to ABSCISIC ACID INSENSITIVE-4: Involvement of abscisic acid in sugar responses. Plant J.

[B29] Tsai FY, Coruzzi GM (1991). Light represses transcription of asparagine synthetase in photosynthetic and nonphotosynthetic organs of plants. Mol Cel Biol.

[B30] Ngai N, Coruzzi GM, Lo Schiavo F, Last RL, Morelli G, Raikhel NV (1998). Dissecting light repression of the asparagine synthetase gene (AS1) in Arabidopsis. Cellular Integration of Signalling Pathways in Plant Development.

[B31] Ngai N (1997). A molecular dissection of DNA elements and transfactors involved in light-induced transcriptional repression of the pea AS1 gene. PhD thesis.

[B32] Li HM, Altschmied L, Chory J (1994). Arabidopsis mutants define downstream branches in the phototransduction pathway. Genes Dev.

[B33] Davidor S (2004). Characterization of putative mutants in Arabidopsis thaliana to determine possible misregulation in light and carbon signaling pathways. Masters thesis.

[B34] Benjamini Y, Hochberg Y (1995). Controlling the false discovery rate: a practical and powerful approach to multiple testing. Journal of the Royal Statistical Society.

[B35] Schoof H, Zaccaria P, Gundlach H, Lemcke K, Rudd S, Kolesov G, Arnold R, Mewes HW, Mayer FX (2002). MIPS *Arabidopsis thaliana *database (MAtDB): an integrated biological knowledge resource based on the first complete plant genome. Nuc Acids Res.

[B36] MatDB. http://mips.gsf.de/proj/plant/jsf/athal/index.jsp.

[B37] GO-Term finder. http://search.cpan.org/~sherlock/GO-TermFinder-0.5/.

[B38] BIOMAPS. http://virtualplant.bio.nyu.edu/cgi-bin/vpweb/virtualplant.cgi.

[B39] Davuluri R, Sun H, Palaniswamy S, Matthews N, Molina C, Kurtz M, Grotewold E (2003). AGRIS: Arabidopsis gene regulatory information server, an information resource of arabidopsis *cis*-regulatory elements and transcription factors. BMC Bioinformatics.

[B40] Yu H, Luscombe NM, Lu HX, Zhu X, Xia Y, Han J-DJ, Bertin N, Chung S, Vidal M, Gerstein M (2004). Annotation transfer between genomes: protein-protein interologs and protein-DNA regulogs. Genome Res.

[B41] Hu G, Chung YL, Glover T, Valentine V, Look AT, Fearon ER (1997). Characterization of human homologs of the Drosophila seven in absentia (sina) gene. Genomics.

[B42] Wei N, Kwok S, von Arnim AG, Lee A, McNellis TW, Piekos B, Deng XW (1994). Arabidopsis *COP8*, *COP10 *and *COP11 *genes are involved in repression of photomorphogenic development in darkness. Plant Cell.

[B43] Chory J, Peto C, Feinbaum R, Pratt L, Ausubel F (1989). Arabidopsis thaliana mutant that develops as a light-grown plant in the absence of light. Cell.

[B44] Nagatani A, Reed JW, Chory J (1993). Isolation and initial characterization of Arabidopsis mutants that are deficient in phytochrome A. Plant Physiol.

[B45] Koornneef M, Rolf E, Spruit CJP (1980). Genetic control of light-inhibited hypocotyls elongation in Arabidopsis thaliana (L.) Heynh. Zeitschrift Fur Pflanzenphysiologie.

[B46] Mockler TC, Guo H, Yang H, Duong H, Lin C (1999). Antagonistic actions of Arabidopsis cryptochromes and phytochrome B in the regulation of floral induction. Development.

[B47] Tepperman JM, Hudson ME, Khanna R, Zhu T, Chang SH, Wang X, Quail PH (2004). Expression profiling of *phyB *mutant demonstrates substantial contribution of other phytochromes to red-light-regulated gene expression during seedling de-etiolation. Plant J.

[B48] Tepperman JM, Zhu T, Chang HS, Wang X, Quail PH (2001). Multiple transcription-factor genes are early targets of phytochrome A signaling. Proc Natl Acad Sci USA.

[B49] Xenarios I, Salwinski L, Duan XJ, Higney P, Kim SM, Eisenberg D (2002). DIP, the database of interacting proteins: a research tool for studying cellular networks of protein interactions. Nucleic Acids Res.

[B50] Kanehisa M, Goto S, Kawashima S, Nakaya A (2002). KEGG: Kyoto encyclopedia of genes and genomes. Nucleic Acids Res.

[B51] Mueller LA, Zhang P, Rhee SY (2003). AraCyc: A biochemical pathway database for arabidopsis. Plant Physiol.

[B52] Alfarano C, Andrade CE, Anthony K, Bahroos N, Bajec M, Bantoft K, Betel D, Bobechko B, Boutilier K, Burgess E, Buzadzija K, Cavero R, D'Abreo C, Donaldson I, Dorairajoo D, Dumontier MJ, Dumontier MR, Earles V, Farrall R, Feldman H, Garderman E, Gong Y, Gonzaga R, Grystan V, Gryz E, Gu V, Haldorsen E, Halupa A, Haw R, Hrvojic A, Hurrell L, Isserlin R, Jack F, Juma F, Khan A, Kon T, Konopinsky S, Le V, Lee E, Ling S, Magidin M, Moniakis J, Montojo J, Moore S, Muskat B, Ng I, Paraiso JP, Parker B, Pintilie G, Pirone R, Salama JJ, Sgro S, Shan T, Shu Y, Siew J, Skinner D, Snyder K, Stasiuk R, Strumpf D, Tuekam B, Tao S, Wang Z, White M, Willis R, Wolting C, Wong S, Wrong A, Xin C, Yao R, Yates B, Zhang S, Zheng K, Pawson T, Ouellette BFF, Houge CWV (2005). The biomolecular interaction network database and related tools 2005 update. Nucleic Acids Res.

[B53] Matys V, Fricke E, Geffers R, GoSzling E, Haubrock M, Hehl R, Hornischer K, Karas D, Kel AE, Kel-Margoulis OV (2003). TRANSFAC: transcriptional regulation, from patterns to profiles. Nucleic Acids Res.

[B54] Shannon P, Markiel A, Ozier O, Baliga NS, Wang JT, Ramage D, Amin N, Schwikowski B, Ideker T (2003). Cytoscape: a software environment for integrated models of biomolecular interaction networks. Genome Res.

[B55] van Helden J, Andre B, Collado-Vides JA Web site for the computational analysis of yeast regulatory sequences. Yeast.

[B56] Caselle M, Di Cunto F, Provero P (2002). Correlating overrepresented upstream cis-regulatory elements to gene expression: a computational approach to regulatory element discovery in eukaryotes. BMC Bioinformatics.

