# Supplementary figures and images for: An integrated genetic, genomic and systems approach defines gene networks regulated by the interaction of light and carbon signaling pathways in Arabidopsis
Source: BMC Syst Biol. 2008 Apr 4;2:31. doi: 10.1186/1752-0509-2-31 (PMC2335094; doi:10.1186/1752-0509-2-31)

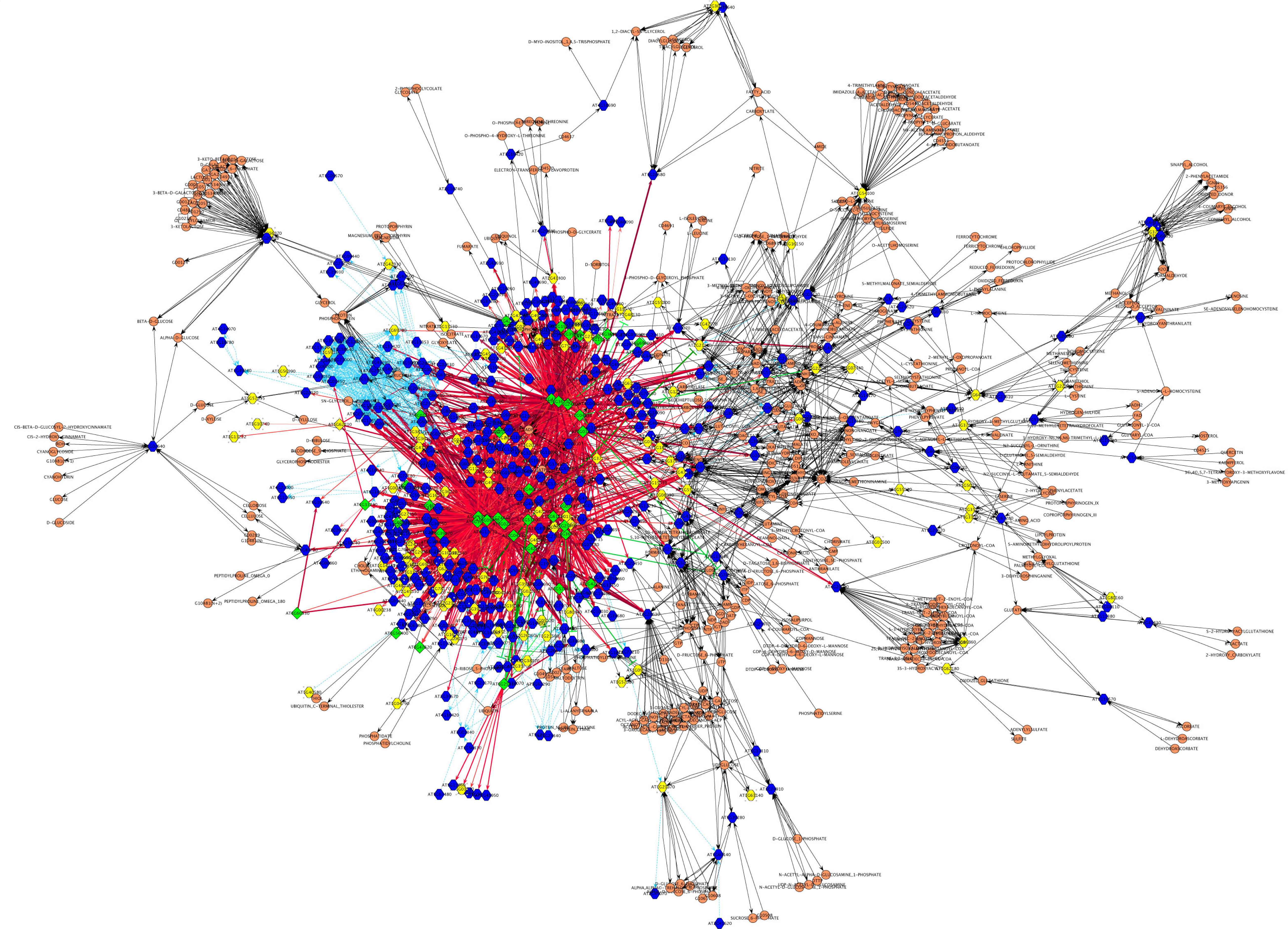

Supplement: Additional file 5 — Multinetwork of L/C regulated genes in wild-type. Metabolic and regulatory network created using the Arabidopsis Multinetwork Tool [21], using the 966 L/C regulated set of genes and visualized with Cytoscape [54]. [file 1752-0509-2-31-S5.jpeg]

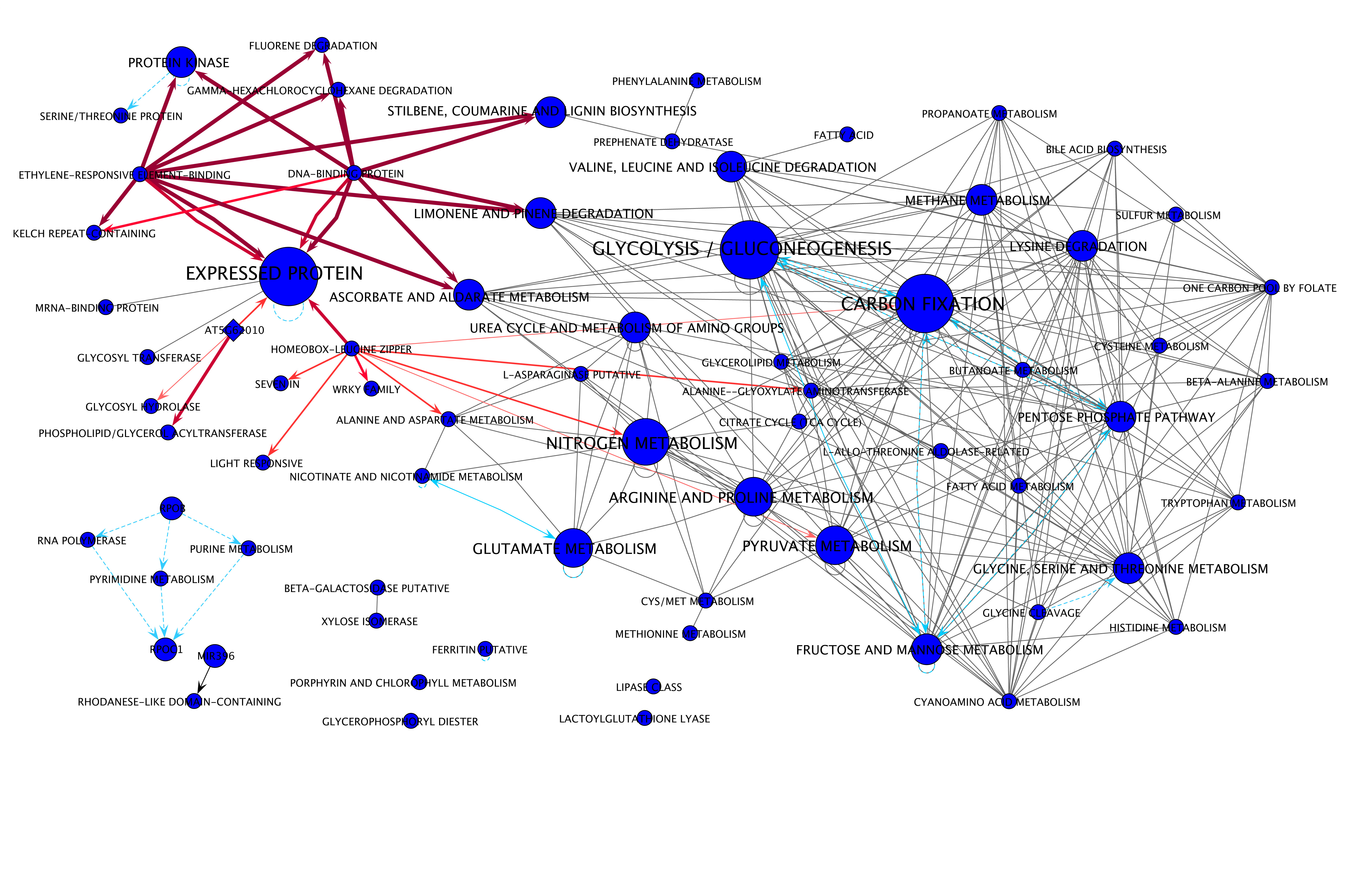

Supplement: Additional file 6 — Supernode network of misregulated genes in cli186. Supernode network created from the 216 misregulated genes in cli186. [file 1752-0509-2-31-S6.jpeg]

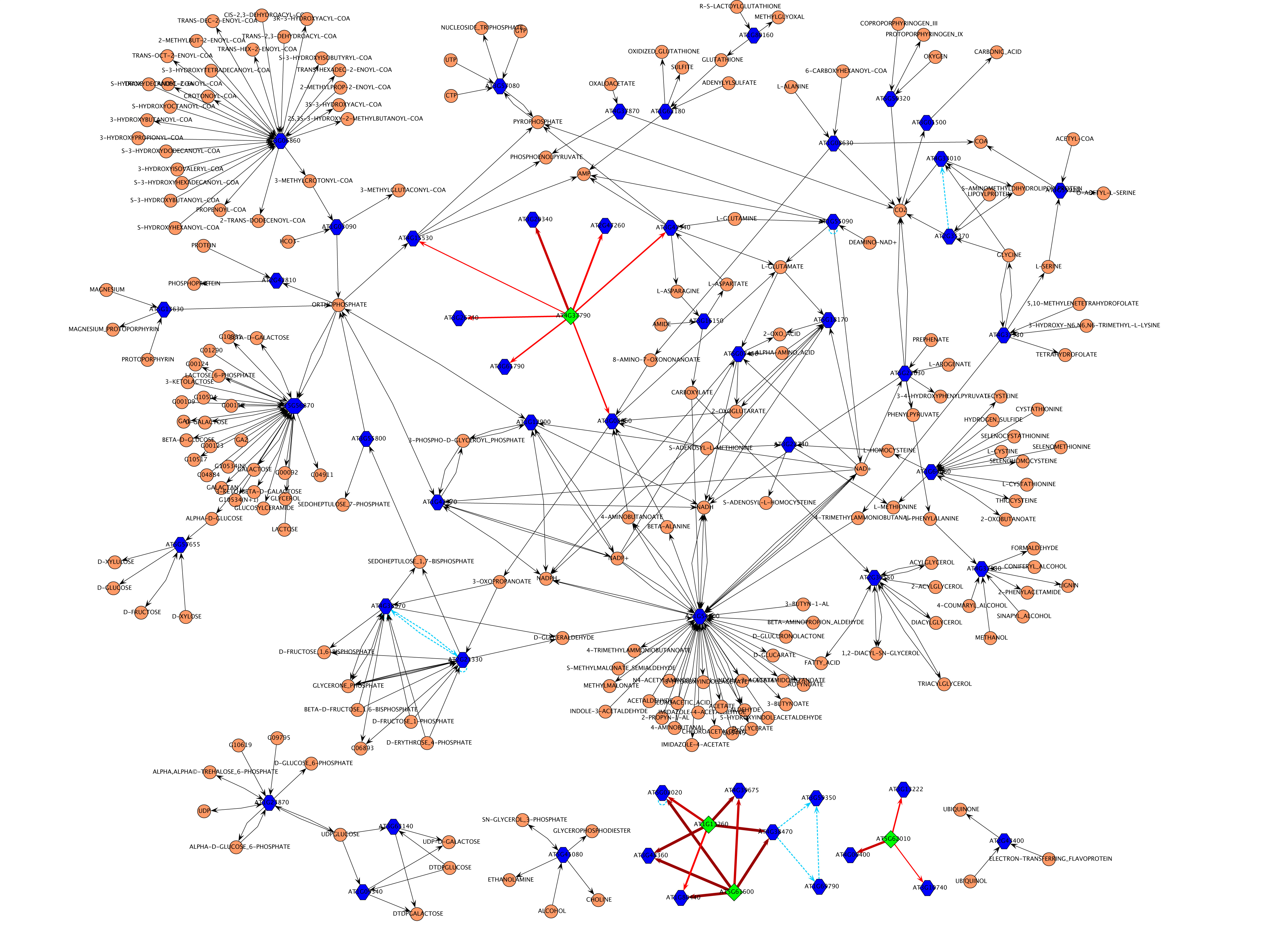

Supplement: Additional file 7 — Misregulated multinetwork in cli186. Metabolic and regulatory network created using the Arabidopsis Multinetwork Tool [21], using the 216 L/C misregulated set of genes from cli186 and visualized with Cytoscape [54]. [file 1752-0509-2-31-S7.jpeg]
