# Supplementary material for: An integrated genetic, genomic and systems approach defines gene networks regulated by the interaction of light and carbon signaling pathways in Arabidopsis
Source: BMC Syst Biol. 2008 Apr 4;2:31. doi: 10.1186/1752-0509-2-31 (PMC2335094; doi:10.1186/1752-0509-2-31)
Supplement: Additional file 9 — Q-PCR validation. q-PCR validation of misregulation of HAT22 and its target genes. [file 1752-0509-2-31-S9.ppt]

## Slide 1
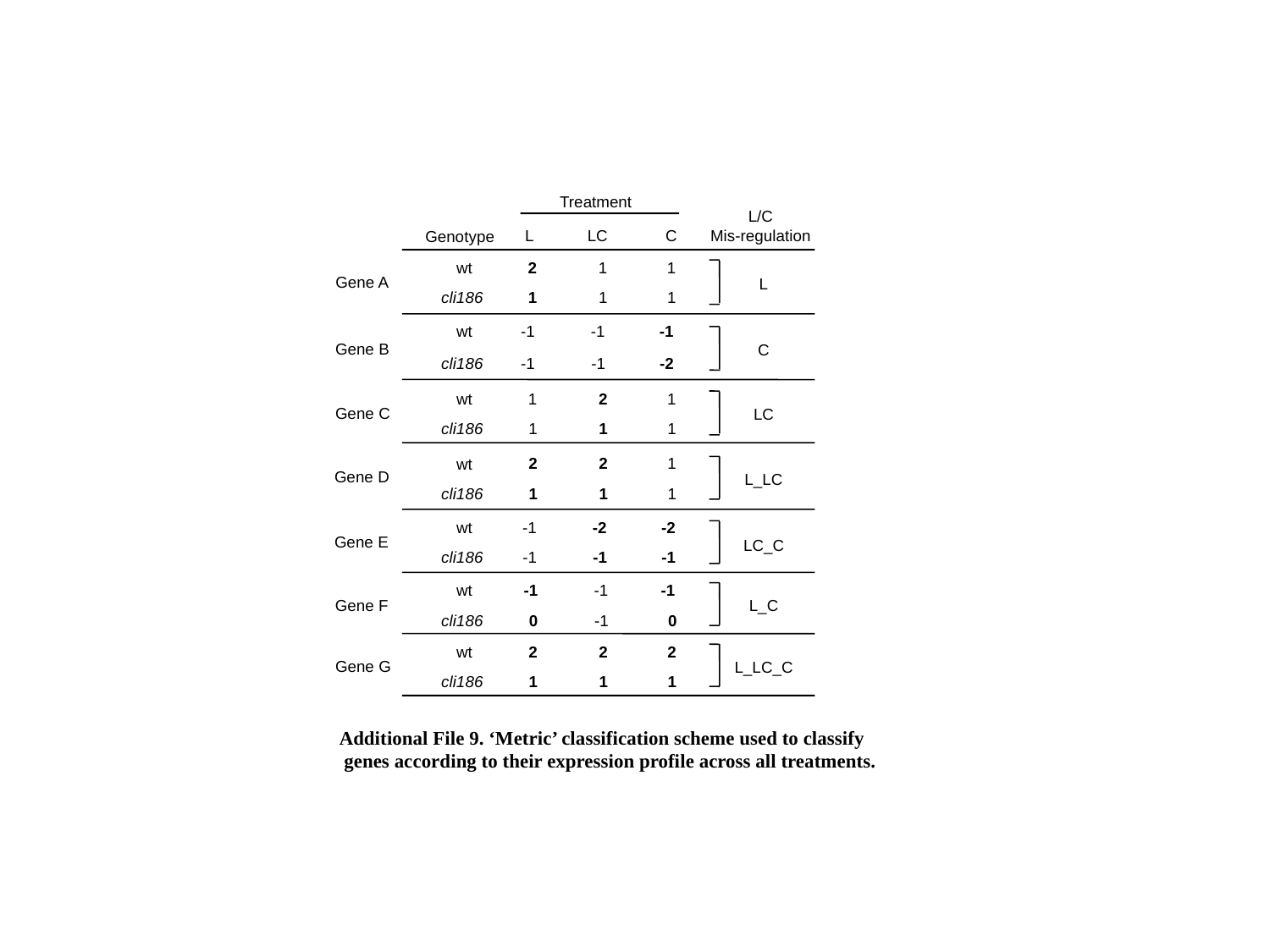

Treatment
 L/C
Mis-regulation
L
LC
C
Genotype
wt
2
1
1
Gene A
L
cli186
1
1
1
wt
-1
-1
-1
Gene B
C
cli186
-1
-1
-2
wt
1
2
1
Gene C
LC
cli186
1
1
1
2
2
1
wt
Gene D
L_LC
1
1
1
cli186
wt
-1
-2
-2
Gene E
LC_C
cli186
-1
-1
-1
wt
-1
-1
-1
Gene F
L_C
cli186
0
-1
0
2
2
2
wt
Gene G
L_LC_C
1
1
1
cli186
Additional File 9. ‘Metric’ classification scheme used to classify
 genes according to their expression profile across all treatments.
